# Optimal transplantation strategy using human induced pluripotent stem cell‐derived cardiomyocytes for acute myocardial infarction in nonhuman primates

**DOI:** 10.1002/mco2.289

**Published:** 2023-06-07

**Authors:** Hong‐mei Li, Ting Wang, Yu‐yin Feng, Ke Sun, Guang‐rui Huang, Yu‐lin Cao, An‐long Xu

**Affiliations:** ^1^ School of Life Science Beijing University of Chinese Medicine Beijing P. R. China; ^2^ Beizhong Jingyuan Biotechnology (Beijing) Limited Beijing P. R. China; ^3^ Tangyi Holdings (Shenzhen) Limited Shenzhen P. R. China; ^4^ State Key Laboratory of Biocontrol Guangdong Province Key Laboratory for Pharmaceutical Functional Genes College of Life Sciences Sun Yat‐Sen University Guangdong P. R. China

**Keywords:** cardioprotection mechanism, efficacy evaluation, nonhuman primates, optimized hiPSC‐CMs, transplant strategies

## Abstract

Cardiomyocytes derived from human induced pluripotent stem cells (hiPSC‐CMs) have the potential to be a therapeutic option for myocardium restoration. However, hiPSC‐CMs of varying maturation and transplantation routes exhibit different reactivity and therapeutic effects. We previously demonstrated that the saponin^+^ compound induces more mature hiPSC‐CMs. The safety and efficacy of multi‐route transplantation of saponin^+^ compound‐induced hiPSC‐CMs in a nonhuman primate with myocardial infarction will be investigated for the first time in this study. Our findings indicate that optimized hiPSC‐CMs transplanted via intramyocardial and intravenous routes may affect myocardial functions by homing or mitochondrial transfer to the damaged myocardium to play a direct therapeutic role as well as indirect beneficial roles via anti‐apoptotic and pro‐angiogenesis mechanisms mediated by different paracrine growth factors. Due to significant mural thrombosis, higher mortality, and unilateral renal shrinkage, intracoronary transplantation of hiPSC‐CMs requires closer attention to anticoagulation and caution in clinical use. Collectively, our data strongly indicated that intramyocardial transplantation of hiPSC‐CMs is the ideal technique for clinical application; multiple cell transfers are recommended to achieve steady and protracted efficacy because intravenous transplantation's potency fluctuates. Thus, our study offers a rationale for choosing a therapeutic cell therapy and the best transplantation strategy for optimally induced hiPSC‐CMs.

## Graphical abstract

This study compared the feasibility, safety, and efficacy of different saponin^+^ compound‐induced cardiomyocytes derived from human induced pluripotent stem cells (hiPSC‐CMs) transplantation strategies on acute MI in nonhuman primates, demonstrating an effective mechanism of optimized hiPSC‐CMs transplantation via intravenous and intramyocardial routes. A subsequent mechanistic study found that optimized hiPSC‐CMs transplanted via intravenous and intramyocardial routes remained in the infarcted and marginal heart regions after 4 weeks, indicating a direct therapeutic role. Besides, sequencing technology was used to examine heart tissue for changes in various pathways and gene expression levels, and a growth factor array was used to detect paracrine factors released in the transplanted macaques' peripheral blood. We systematically assessed the efficacy and safety of various hiPSC‐CMs transplantation routes in the treatment of MI and identified a potential mechanism, providing scientific evidence to guide clinicians in selecting an individualized hiPSC‐CMs transplantation strategy.

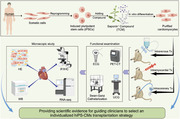

## 1 INTRODUCTION

Myocardial infarction (MI) is a leading cause of death and disability worldwide,^1^ accounting for approximately 30% of all deaths globally.^2^ Although mechanical reperfusion by percutaneous coronary intervention has reduced the acute mortality rates of MI, the rehospitalization for heart failure rates in patients with larger MI remain as high as 15%–35%.^3^ As the heart lacks the regenerative potential to replace the dramatic cell loss that occurs post‐MI, numerous stem cell therapies have been intensively studied, especially the use of cardiomyocytes derived from human induced pluripotent stem cells (hiPSC‐CMs) to treat MI, which represents a promising cell source for cardiac cell therapy.^4^, ^5^

Currently, the main routes of hiPSC‐CMs transplantation are intravenous injection, intracoronary injection, and direct intramyocardial injection. An intramyocardial injection is the most effective route for precise cell delivery during open‐chest revascularization surgery, while in monkeys, hiPSC‐CMs have been found to cause a transient period of ventricular arrhythmias.^6^ Intravenous transplantation is almost noninvasive; however, the majority of cells may migrate to other organs and few may remain in the infarct site.^7^ For patients undergoing percutaneous infarct‐associated arterial revascularization after acute MI, infusion of transplanted cells through intracoronary injection is a feasible method; however, most of the transplanted cells are cleared in a short period of time,^7^ and the results of efficacy and safety studies are inconsistent.^8^, ^9^, ^10^, ^11^ One cause of these issues is that the transplanted cells are not mature enough, and the other is that the route of transplantation impacts the healing outcome.

In view of the insufficient maturity of transplanted cells, our group has developed a method for hiPSC‐CMs formation and functional maturation by adding a uniquely prepared compound (mainly comprised of saponin and other well‐defined small molecules) based on our previous research.^12^, ^13^ The saponin^+^ compound‐induced hiPSC‐CMs are highly consistent with the physiological characteristics of the late stage of fetal heart development and have a more mature structure, stronger drug sensitivity, more stable electrophysiological conduction function, and better therapeutic effects in mice.^13^ However, the most appropriate transplantation method for saponin^+^ compound‐induced hiPSC‐CMs is unclear. To further investigate the potential of the optimized hiPSC‐CMs for in vivo use in physiologically relevant large animals, we selected the nonhuman primate, *Macaca rhesus*, due to its evolutionary proximity to humans and the similarity of the two species’ cardiovascular systems. The present study aimed to thoroughly compare the therapeutic effects of optimally generated hiPSC‐CMs for transplantation via different routes to the macaques with acute MI to address the following outcomes: (1) the cell retention, direct repair potential, and paracrine function in the MI heart after hiPSC‐CMs transplantation; (2) the impact on cardiac remodeling and heart function posttransplantation; and (3) the benefits and shortcomings of hiPSC‐CMs transplantation by different strategies.

Here, we compared the viability, safety, and efficacy of various transplantation strategies of saponin^+^ compound‐induced hiPSC‐CMs on acute MI in nonhuman primates, demonstrating that hiPSC‐CMs transplanted by intramyocardial and intravenous routes retained to the infarcted and marginal heart regions within 4 weeks, and mitochondrial transfer might be one of the factors that produced therapeutic effects. Additionally, sequencing technology was applied to analyze heart tissue for changes in various pathways and gene expression levels, and a growth factor array was utilized to detect the paracrine factors released in the peripheral blood of the transplanted macaques. Together, we systematically assessed the efficacy and safety of different hiPSC‐CMs transplantation routes in the treatment of MI and identified a potential mechanism, providing scientific evidence for guiding clinicians to select an individualized hiPSC‐CMs transplantation strategy.

## 2 RESULTS

### 2.1 Preparation and identification of saponin^+^ compound‐induced hiPSC‐CMs

Nanog, Oct‐4, Sox‐2, and SSEA‐4 were found to be strongly expressed in more than 95% of the cells when we tested the pluripotency of hiPSC at Day 0 before differentiation (Figure S1A). Based on the theoretical underpinnings of cardiac embryonic stage development and prior research methodologies,^13^, ^14^ we integrated an enhanced differentiation process called the “compound induction scheme.” We first used CHIR99021 to block the GSK3β signaling pathway, then bFGF, IWP2, and finally our own saponin^+^ compound to stimulate the creation of mesoderm and encourage cell differentiation into contracting cardiomyocytes (Figure S1B). On Day 15, once the cells had begun to contract, the expression of cardiac‐specific proteins in optimally differentiated hiPSC‐CMs was assessed. The cardiac‐specific proteins c‐TNI, TNNT‐2, and α‐actinin were expressed by almost all hiPSC‐CMs (Figure S1C).

### 2.2 Optimized hiPSC‐CMs transplantation improves cardiac function, attenuates intracardiac pressure, and restores hemodynamic balance

**FIGURE 1:**
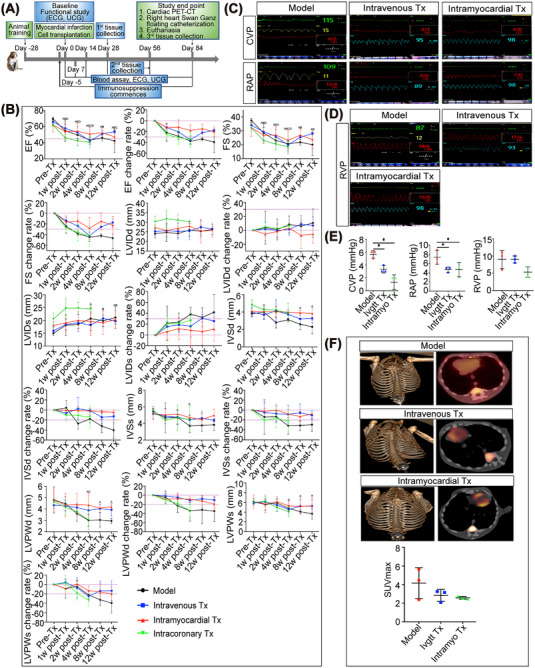
Effects of the saponin^+^ compound‐induced hiPSC‐CMs transplanted through different routes on cardiac function and myocardial energy metabolism. (A) Timeline for the efficacy study of the hiPSC‐CMs transplantation experiments in myocardial infarction model of nonhuman primates. Echocardiography and cardiac catheterization were performed to assess cardiac function. The changes in myocardial energy metabolism were tracked by cardiac PET‐CT. Electrocardiogram (ECG) was also included to monitor the occurrence of arrhythmias. (B) Cardiac dimensions and function of macaque hearts were investigated in vivo by echocardiography at different time points (before transplantation and 1, 2, 4, 8, and 12 weeks posttransplantation). Echocardiographic data, including EF, FS, LVIDd, LVIDs, IVSd, IVSs, LVPWd, and LVPWs values, were collected and analyzed. EF, ejection fraction; FS, fractional shortening; LVIDd, left ventricular internal diastolic dimension; LVIDs, left ventricular internal systolic dimension; IVSd, interventricular septal thickness at diastole; IVSs, interventricular septal thickness at systole; LVPWd, left ventricular posterior wall dimension in diastole; LVPWs, left ventricular posterior wall dimension in systole. *n* = 3–5 macaques per group. ^A^ indicates *P* < 0.05 post‐Tx versus pre‐Tx in the model group. ^B^ indicates *p* < 0.05 post‐Tx versus pre‐Tx in the intravenous transplantation group. ^C^ indicates *p* < 0.05 post‐Tx versus pre‐Tx in the intramyocardial transplantation group.^D^ indicates *p* < 0.05 post‐Tx versus pre‐Tx in the intracoronary transplantation group. (C–E) Right heart catheterization was performed using a balloon‐tipped thermodilution 4F Swan‐Ganz catheter for measurements of hemodynamic variables and cardiac cavity pressure, including central venous pressure (CVP), right atrial pressure (RAP), and right ventricular pressure (RVP). ECG waveform and heart rate are represented by the green line and value, respectively. The yellow line and value represent the respiratory waveform and respiratory rate. The blue line and value represent the pulse oxygen waveform and blood oxygen value. The red line and values represent the intracardiac pressure waveform as well as the systolic, diastolic, and mean pressure values. Model group, intravenous Tx group, and intramyocardial Tx group, with 3 macaques in each group. The intracoronary Tx group did not have the safety of application, so it was not included in the follow‐up effectiveness evaluation study. (F) Myocardial metabolism was assessed via ^18^F‐DPA‐714 uptake by PET/CT. Representative 3D reconstruction images of PET were transferred to a Siemens‐dedicated workstation to obtain PET/CT fusion images. Reconstructed slice thickness: 5 mm. The higher glucose uptake level was displayed as the increase in the intensity of red as indicated in each image. Calculation of the maximum standard uptake value (SUVmax) showed that both the intravenous and intramyocardial transplantation groups had a trend of decreasing glucose uptake levels after 12 weeks of hiPSC‐CMs treatment (*n*  =  3 macaques per group).

For cardiac functional studies, we induced acute myocardial infarcts by occluding the mid‐LAD for 3 h followed by reperfusion. Immunosuppressive agents were administered starting 5 days before hiPSC‐CMs transplantation (defined as Tx), and cells were injected on the day of post‐infarction via intravenous, intramyocardial and intracoronary routes (Figure 1A). Transthoracic M‐mode echocardiography was performed at normal baseline (pre‐MI/Tx) and 1, 2, 4, 8, and 12 weeks post‐Tx. The MI model group showed a significant progressive downward trend in ejection fraction (EF) and fractional shortening (FS) during the follow‐up period. Both the intravenous and intramyocardial Tx groups showed relative improvement in EF and FS change rates compared to the model group. In comparison, the improvement of EF and FS change rates in the intramyocardial Tx group was more stable, while the improvement effect of the intravenous group fluctuated, especially at 4 weeks post‐Tx. In particular, EF and FS decreased significantly in the intracoronary Tx group compared to the model group, and two of the three macaques died 4 weeks after intracoronary transplantation. The only surviving macaque in the intracoronary Tx group was in poor condition and could not tolerate subsequent functional tests. Then, we observed structural deterioration in the model group with a significant decrease in the interventricular septal thickness at diastole (IVSd), interventricular septal thickness at systole (IVSs), left ventricular posterior wall dimension in diastole (LVPWd), and left ventricular posterior wall dimension in systole (LVPWs), which was accompanied by a relative increase in left ventricular internal diastolic dimension (LVIDd) and left ventricular internal systolic dimension (LVIDs) post‐modeling (compared to pre‐MI/Tx baseline). However, the structure was almost preserved in animals treated with hiPSC‐CMs via the intramyocardial transplantation route. Echocardiographic analysis revealed that IVSd, IVSs, and LVPWd remained steady, and no obvious downward trend was observed in the intravenous Tx group. However, the improvement effect on LVIDd, LVIDs, and LVPWs was not obvious as the indices fluctuated after 4 weeks of intravenous transplantation. Unlike intravenous and intramyocardial Tx groups, intracoronary transplantation of hiPSC‐CMs had a little protective effect on myocardial structural remodeling and even partially worsened the deterioration of cardiac structure (Figure 1B). These results indicated that intramyocardial Tx had better capacity with respect to the restoration of cardiac function in infarcted macaque hearts compared to the intravenous Tx group, while the intracoronary transplantation route had a greater risk of exacerbating cardiac disease to cause death.

The changes in intracardiac pressure were assessed by measuring central venous pressure (CVP), right atrial pressure (RAP), and right ventricular pressure (RVP) via right heart catheterization to evaluate the in vivo hemodynamic state (Figure 1C–E). There was no difference in the mean levels of CVP, RAP, and RVP between the intravenous Tx group and the intramyocardial Tx group (CVP: 3.33 ± 0.58 vs. 1.33 ± 1.15; RAP: 4.67 ± 5.58 vs. 4.67 ± 1.53; RVP: 9.00 ± 1.00 vs. 5.33 ± 1.53; *p* > 0.05). However, the levels of CVP and RAP were significantly reduced in the intravenous and intramyocardial Tx groups compared to the model group (CVP: 5.67 ± 0.58; RAP: 7.33 ± 1.53; *p* < 0.05). The macaques in the intracoronary transplantation group were not tested for intracardiac pressure for two of three macaques failed to survive to the end of the experiment, the only animal that survived could not tolerate invasive examination. The above parameters indicated that the optimized hiPSC‐CMs transplantation improved systolic and diastolic performances of the right heart. Because there is no matching left ventricular catheter, and macaques have poor tolerance and a small heart, the left ventricular intraluminal pressure cannot be measured.

### 2.3 Optimized hiPSC‐CMs transplantation has the potential to inhibit myocardial energy reconstruction in macaques with MI

^18^F‐FDG PET‐CT scanning was performed at 12 weeks post‐Tx to determine the effect of hiPSC‐CMs on viable myocardium metabolism (Figure 1F). In general, normal myocardium utilizes fatty acid metabolism rather than glucose metabolism. Once MI occurs, glucose metabolism is feedback‐activated for energy reconstruction. Compared to the substantial uptake of ^18^F‐FDG in the model group, a decreasing trend of myocardial maximum standard uptake value (SUVmax) occurred in the intravenous and intramyocardial Tx groups (2.84 ± 0.65 in the intravenous Tx group and 2.58 ± 0.15 in the intramyocardial Tx group, vs. 4.16 ± 1.65 in the model group; *p* > 0.05). Collectively, these data suggested that both the intravenous and intramyocardial transplantation of hiPSC‐CMs might have the potential to restore cardiac metabolism in a macaque model of MI injury.

### 2.4 Safety evaluation of transplantation of saponin^+^ compound‐induced hiPSC‐CMs via different routes

To evaluate the effect of transplanted hiPSC‐CMs on the electrophysiological function of the infarcted hearts, we performed an electrocardiogram (ECG) in a nonhuman primate at different time points. Typical ST elevations were observed in all macaques posttransplantation, but no macaque in any transplantation group developed persistent malignant arrhythmia at specific time points (Figure S3A–D). Interestingly, obvious sinus bradycardia occurred at the 8th‐week post‐Tx via intramyocardial injection, but the heart rate returned to baseline at week 12 post‐Tx. No significant deterioration of ECG was observed in the intracoronary transplantation group, but two of the three macaques died 4 weeks post‐transplantation.

**FIGURE 2:**
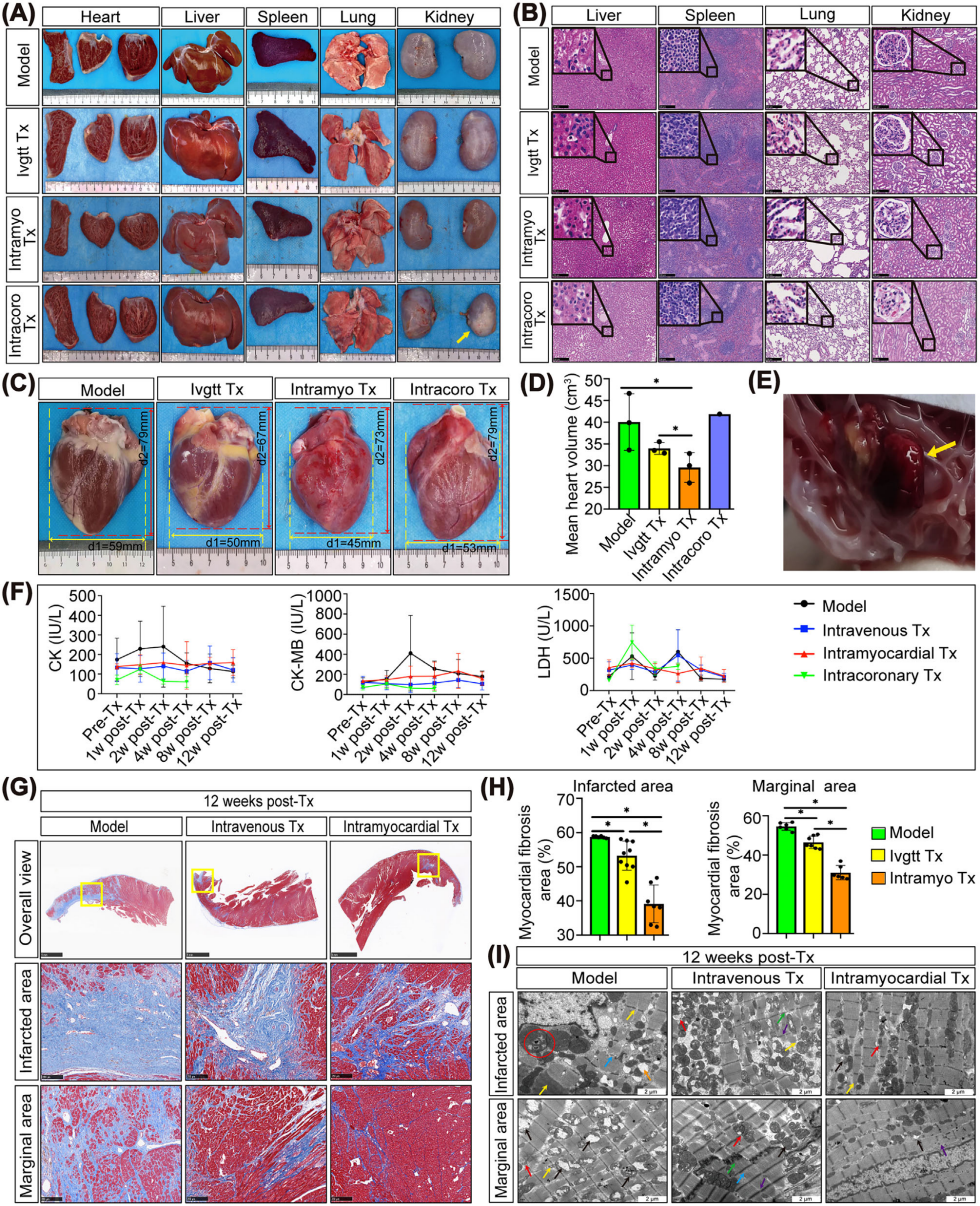
Safety evaluation and analysis of the transplantation of saponin^+^ compound‐induced hiPSC‐CMs on cardiac structure and secretion of myocardial enzymes. (A) Preliminary safety evaluation by macroscopic comparison of important organ morphology. One of the kidneys showed atrophy and sclerosis (yellow arrow) after intracoronary transplantation of hiPSC‐CMs. No obvious abnormalities were found in the other groups. (B) Tissue sections were stained with H&E for pathological examination. Microscopic analysis revealed no evidence of tumor formation after hiPSC‐CM transplantation. Scale bars = 250 μm. (C, D) The comparison of heart anatomy showed significantly smaller sizes of the heart in the intramyocardial transplantation group than in the model group. * indicates *p* < 0.05. *n* = 3 macaques for the model, intravenous and intramyocardial group. There was only one of three macaques that lived to the terminal time point in the intracoronary group. (E) Anatomical examination of hearts in macaques after transplantation via the intracoronary route showed mural thrombi. The yellow arrow refers to the formed mural thrombus. (F) Effects of hiPSC‐CM transplantation on the levels of CK and its isoenzymes, namely, CK‐MB and LDH, in macaques with myocardial infarction. (G) The extent of fibrosis (blue‐stained area) was examined by staining paraffin‐embedded heart sections using Masson's trichrome staining. The overall view image has a scale bar of 5 mm, whereas the images of the infarcted and marginal areas have a scale bar of 250 μm. (H) Fibrosis was assessed by semi‐quantification of Masson trichrome‐positive tissue with the Image‐Pro plus analysis system. * indicates *p* < 0.05. (I) Ultrastructural changes in the myocardium of the model and hiPSC‐CM‐transplanted groups were revealed by transmission electron microscope analysis at high (scale bar = 2 μm) magnifications. Black arrow indicates mitochondrial crest and membrane structure. Red arrow indicates autophagy of mitochondria. Yellow arrow indicates myofilament and sarcomere. Blue arrow indicates myocardial fiber. Purple arrow indicates the Z‐line. Green arrow indicates myocardial fiber. Green arrow indicates the structure of the intercalated disc. Orange arrow indicates sarcoplasmic reticulum. Red circle indicates myelin‐like structures and glycogen particle deposition. Model: MI‐model group that received no cell transplantation; Ivgtt Tx, intravenous transplantation group; Intramyo Tx, intramyocardial transplantation group; Intracoro, intracoronary transplantation group.

Further, an experienced primate pathologist collected the heart, liver, spleen, lung, and kidney from each group of macaques for a detailed gross and microscopic examination. No teratoma was observed 12 weeks after the intravenous and intramyocardial transplantation of hiPSC‐CMs, supporting the safety of potential therapy. Notably, we found unilateral renal atrophy in the intracoronary transplantation group (yellow arrow), while no obvious pathological abnormalities were observed in the atrophic kidney (Figure 2A,B). In addition, we observed a significant decrease in cardiac morphology at 12 weeks after intramyocardial transplantation of hiPSC‐CMs (*p* < 0.05), while the heart volume in the intravenous and intracoronary Tx groups did not change significantly compared to that in the model group (Figure 2C,D). Surprisingly, autopsies demonstrated typical mural thrombosis in the hearts of the intracoronary Tx group (yellow arrow), which was speculated to be the cause of animal death after hiPSC‐CMs transplantation (Figure 2E). Moreover, peripheral blood was collected to detect liver and kidney function indicators, which showed no obvious liver or kidney damage (Figure S4A), and there was no significant difference in the weight of important organs compared to the model group (Figure S4B), confirming the safety of the intravenous and intramyocardial transplantation of hiPSC‐CMs.

### 2.5 Myocardial enzyme detection and structural analysis after hiPSC‐CMs transplantation

Within 4 weeks post‐Tx, the expression of CK and CK‐MB in all the transplantation groups was relatively stable, and there was no rapid increase in myocardial enzymes as in the model group. With the exception of LDH expression in the intramyocardial Tx group remaining stable, the other groups showed large fluctuations in LDH expression, especially at 1 and 4 weeks post‐Tx (Figure 2F).

Cardiac remodeling was further examined by Masson's trichrome staining. The intravenous and intramyocardial transplantation of hiPSC‐CMs led to a decrease in adverse cardiac remodeling with reduced fibrosis in the infarcted area (intravenous Tx: 53.26 ± 4.27; intramyocardial Tx: 39.13 ± 5.51; *p* < 0.05) and marginal area (intravenous Tx: 46.48 ± 3.14; intramyocardial Tx: 31.04 ± 3.61; *p* < 0.05) compared to the model group (Figure 2G,H). Compared to the intravenous Tx group, the effect of reversing myocardial fibrosis was more obvious in the intramyocardial Tx group (*p* < 0.05).

The ultrastructure of the myocardium was analyzed by transmission electron microscopy (Figure 2I). The infarcted area of the MI model heart had the following morphology: myocardial fiber rupture and dissolution (blue arrow); severe expansion of sarcoplasmic reticulum (orange arrow), myelin‐like structures, and glycogen particle deposition (red circle); myofilament rupture and deepened color of some sarcomeres (yellow arrows). In the marginal area of the MI model heart, the mitochondrial cristae were broken, forming electron transparent areas and the remaining mitochondrial cristae were disorderly arranged (black arrows). In addition, autophagy (red arrow) and myofilament lysis (yellow arrow) were observed. After intravenous transplantation, a close arrangement of myocardial fibers (blue arrow) and clear mitochondrial crest structure (black arrow) were observed in the marginal area with occasional autophagy of mitochondria and double membrane structure (red arrow). No rupture of the intercalated disc (green arrow) or Z‐line (purple arrow) was observed after intravenous transplantation. After intravenous transplantation, mitochondrial membranes and cristae were broken, and autophagy (red arrow), myofilament lysis (yellow arrow), and Z‐line fracture (purple arrow) were visible in the infarcted area. Moreover, the structure of the intercalated disc was unclear (green arrow) after intravenous transplantation. At 12 weeks post‐intramyocardial transplantation of hiPSC‐CMs, mitochondrial membrane rupture (black arrow) was occasionally observed in the marginal area, and fibers were orderly arranged. In addition, the myofilaments and sarcomeres were almost complete and a small amount of Z‐line rupture (purple arrow) remained after intramyocardial transplantation. In the infarcted area, the myocardial fibers were closely arranged with occasional mitochondrial membrane rupture (black arrow), and autophagy of a few mitochondria was observed (red arrow). In addition, several myofilaments and sarcomere were ruptured (yellow arrow) after intramyocardial transplantation. Overall, the entire ultrastructure was improved to some extent in the intravenous and intramyocardial Tx groups compared to the model group, but the therapeutic effect of the intramyocardial Tx group was better than that of the intravenous Tx group.

### 2.6 Optimized hiPSC‐CMs transplantation modulates mRNA expression profiles in myocardial tissues

**FIGURE 3:**
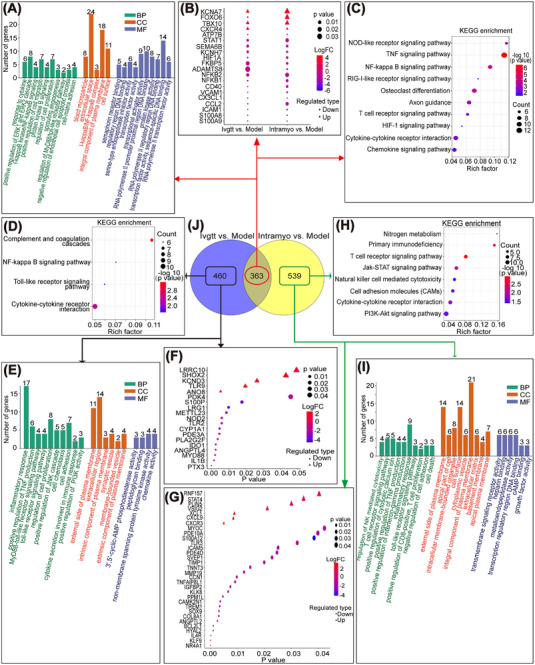
RNA‐Seq technology was used to sequence the transcriptomes of heart tissues in the model and hiPSC‐CM‐treated groups 12 weeks after transplantation. (A–C) The intravenous and intramyocardial transplantation groups were compared to the model group, and the common parts of the differentially expressed genes were analyzed by GO and KEGG. (D–F) Among the differentially expressed genes in the intravenous and intramyocardial transplantation groups compared to the model group, the differentially expressed genes unique to the intravenous group but not to the intramyocardial group were analyzed by GO and KEGG analyses. (G–I) Among the differentially expressed genes in the intravenous and intramyocardial transplantation groups compared to the model group, the differentially expressed genes unique to the intramyocardial group but not to the intravenous group were analyzed by GO and KEGG analyses. (J) Venn diagrams of genes differentially expressed in the two comparisons. The number of overlapping genes > 2‐fold differentially regulated (*p* < 0.001) was determined and mapped. 3 macaques per group. Ivgtt versus Model differentially expressed genes in the hiPSC‐CM intravenous transplantation group after 12 weeks compared to the model group; Intramyo versus Model, differentially expressed genes in the hiPSC‐CM intramyocardial transplantation group after 12 weeks compared to the model group. BP, biological process; CC, cellularcomponent; MF, molecular function.

To investigate the beneficial effects after hiPSC‐CMs transplantation, we performed RNA sequencing. Venn diagrams constructed with gene sets that were significantly altered (Q < 0.05) in the intravenous and intramyocardial transplantation groups identified 363 common differentially expressed genes compared to the model group (Figure 3J). Among the differentially expressed genes, 20 genes (5 upregulated and 15 downregulated) were selected (Figure 3B). Gene ontology (GO) enrichment and Kyoto Encyclopedia of Genes and Genomes (KEGG) enrichment analyses predicted the potential functions of the upregulated and downregulated genes when comparing the intravenous and intramyocardial transplantation groups to the model group. GO enrichment analysis showed an overrepresentation of positive regulation of ERK1 and ERK2 cascade, cell proliferation, and migration, response to cytokine, sprouting angiogenesis, negative regulation of cell apoptosis process, and NF‐κB/MyD88 signaling pathway (Figure 3A). In addition, KEGG analysis indicated that the most downregulated genes were enriched in the NOD pathway, TNF pathway, NF‐κB pathway, and RIG‐1 pathway, whereas the upregulated genes were primarily enriched in the cytokine‐cytokine receptor interaction and HIF‐1 signaling pathway (Figure 3C). We further analyzed the 460 differentially expressed genes unique to the intravenous group but not in the intramyocardial group by GO and KEGG analyses, and 19 genes (five upregulated and 14 downregulated) were selected (Figure 3F). GO enrichment analysis showed the regulation of inflammatory responses, cell proliferation, cell chemotaxis, and adhesion (Figure 3E). KEGG analysis indicated that the most downregulated genes were enriched in the NF‐κB and Toll‐like receptor pathways, whereas the upregulated genes were mainly enriched in cytokine‐cytokine receptor interactions (Figure 3D). Moreover, we further analyzed the 539 differentially expressed genes unique to the intramyocardial group but not in the intravenous group by GO and KEGG analyses, and 32 genes (seven upregulated and 25 downregulated) were selected (Figure 3G). Among the listed GO terms (*p* < 0.05), we were interested in the relatively specific GO terms, we were interested in, included regulation of inflammatory response, positive regulation of cytosolic calcium ion concentration, cell death, and growth factor activity (Figure 3I). KEGG analysis reflected the regulation of the JAK‐STAT pathway, PI3K‐Akt pathway, T cell receptor pathway, cell adhesion molecules, and cytokine‐cytokine receptor interaction (Figure 3H). Together, these data demonstrated that intravenous and intramyocardial transplantation of hiPSC‐CMs partially reversed heart injury by attenuating apoptosis, decreasing inflammation, responding to cytokines, and enhancing angiogenesis.

### 2.7 Different routes of hiPSC‐CMs transplantation reduce cardiomyocyte apoptosis and stimulate Nkx2.5 and GATA4 expression after MI injury

**FIGURE 4:**
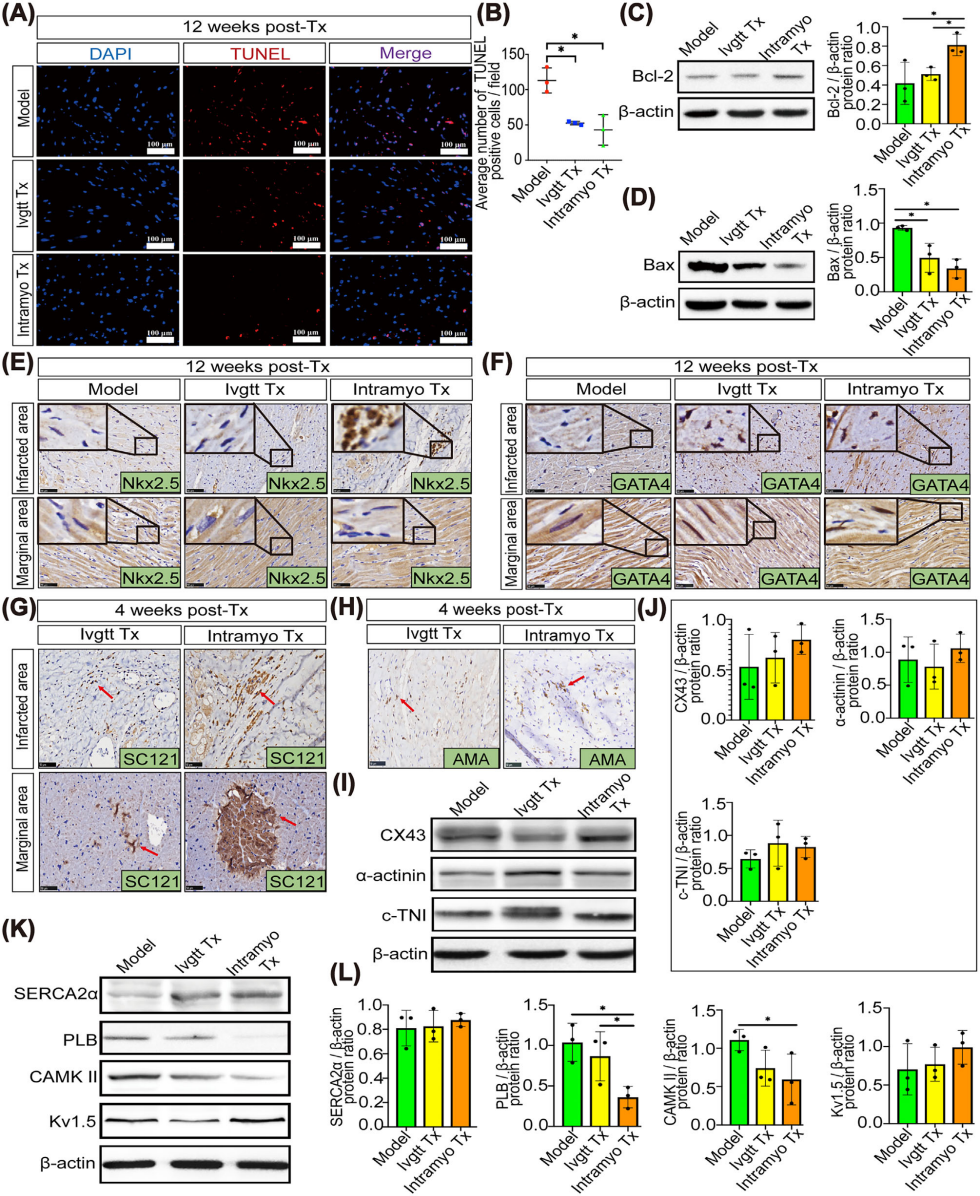
Maturation and integration of transplanted hiPSC‐CMs into monkey hearts reduce host cardiomyocyte apoptosis and enhance endogenous cell survival. (A) A TUNEL assay was used to detect apoptotic cells in the marginal area of infarcted monkey hearts in the model and hiPSC‐CM transplantation groups. Apoptotic nuclei were stained red, and normal nuclei were stained blue. Scale bar = 100 μm. (B) ImageJ software was used to count and statistically analyze the TUNEL‐positive cells. (C, D) The expression levels of Bcl‐2 and Bax were determined by Western blot analysis and normalized to β‐actin. (E, F) Immunohistochemical staining for the Nkx2.5 and GATA4 transcription factors in cardiac tissue sections after 12 weeks of hiPSC‐CM transplantation treatment. The immunopositive areas are stained brown, and the nuclei of negative areas are stained blue with hematoxylin. Scale bar = 50 μm. (G) Immunohistochemical staining of human‐specific cytoplasm (SC121) revealed robust homing of hiPSC‐CMs in the infarcted and marginal areas. Grafts were studied at weeks 4, 8, and 12 posttransplantation. Positive signals resulted in brown staining. Red arrow indicates positive human cells. Scale bar = 50 μm. (H) Human mitochondria were found on tissue sections with positive SC121 results using immunohistochemical labeling with anti‐mitochondrial antibody (AMA) specific to humans in the fourth week following transplantation. Brown staining results from positive signals. Red arrow indicates positive human mitochondria. Scale bar = 50 μm. (I‐J) Western blot analysis of the expression of cardiac proteins related to contractility and gap junctions, including sarcomeric α‐actinin, cardiac troponin I (c‐TnI), and connexin 43 (CX43), in heart tissues at week 12 post‐Tx. Protein expression levels were normalized to β‐actin levels. (K, L) Western blot analysis was used to detect the expression of myocardial calcium handling proteins in heart tissues at week 12 post‐Tx, including sarcoplasmic reticulum Ca^2+^‐ATPase2a (SERCA2a), phospholamban (PLB), calmodulin‐dependent protein kinase II (CAMK II) and potassium channel proteins, such as the mammalian voltage‐dependent potassium channel Kv1.5. Protein expression levels were normalized to β‐actin levels. 3 macaques per group. All data were represented as the mean ± SD. ^*^ *p* < 0.05 was considered significant. Model: MI‐model group that received no cell transplantation; Ivgtt Tx, intravenous transplantation group; Intramyo Tx, intramyocardial transplantation group.

Because RNA‐seq indicated that the common effect of intravenous and intramyocardial transplantation of hiPSC‐CMs was the inhibition of cardiomyocyte apoptosis, we performed a TUNEL assay. Compared to the model group, the number of TUNEL‐positive cardiomyocyte nuclei significantly decreased in the intravenous and intramyocardial Tx groups (Figure 4A). Quantitative statistical analysis of the apoptotic cells demonstrated that there was a marked decrease in the hiPSC‐CMs transplantation groups (Figure 4B). Furthermore, the expression levels of the pro‐apoptotic protein, Bax, and the anti‐apoptotic protein, Bcl‐2, were determined by western blot (WB) assay. Compared to the model group, the intramyocardial Tx decreased Bax expression and increased Bcl‐2 expression, while the intravenous Tx decreased Bax expression but did not significantly affect Bcl‐2 expression (Figure 4C,D). Notably, injured cardiomyocytes expressing Nkx2.5 were found in the infarcted area of the macaque heart after intramyocardial Tx (Figure 4E), which has been shown to play a critical role in repairing the adult heart.^15^ No significant positive expression of Nkx2.5 in the infarcted area of the macaque heart was found in the model and intravenous groups. In addition, GATA4 expression was upregulated after intravenous and intramyocardial transplantation in infarcted and marginal areas of the injured heart (Figure 4F), suggesting that GATA4 overexpression may be activated by hiPSC‐CMs transplantation in the event of MI, which can act in concert to promote myocardial capillarization and heart function.^16^

### 2.8 In situ survival after different routes of hiPSC‐CMs transplantation and its effect on myocardial electrical and structural remodeling

We obtained LV myocardial specimens after transplantation to confirm the survival and homing of the transplanted hiPSC‐CMs. We performed immunohistochemical staining for the human‐specific cytoplasmic marker, SC121, in the infarcted and marginal areas of the macaque heart (Figure 4G). The infarcted and marginal areas had a limited number of intravenously transplanted hiPSC‐CMs with a dispersed distribution, however the positive tagged cells had an uneven form and seemed to be extruded cells or fragments. In the intramyocardial Tx group, we noticed intercellular aggregation at 4 weeks post‐Tx, which suggested that there might be interconnections, as well as scattered positive human cells with a partial agglomeration pattern in the infarct area and a close distribution of lumpy positive cells in the marginal area. Nevertheless, at 8 and 12 weeks following cell transplantation, we were unable to detect the survival of transplanted hiPSC‐CMs in situ. In the further study, we used the specific human mitochondrial antibody AMA for immunohistochemical staining on serial sections of SC121‐positive myocardium taken 4 weeks after transplantation and found human mitochondria, implying that mitochondrial transfers may be one of the potential mechanisms to promote myocardial repair (Figure 4H). To determine the effect of transplanted hiPSC‐CMs on myocardial structural remodeling at week 12 post‐Tx, we detected the CX43 gap junction protein and the expression of the α‐actinin and c‐TNI cardiac markers (Figure 4I). There was a trend for c‐TNI, α‐actinin, and CX43 levels to be increased in the intramyocardial transplantation groups than those in the model group (Figure 4J). In addition, we explored the effect of hiPSC‐CMs transplantation at week 12 post‐Tx on myocardial electrical remodeling to reveal the possible mechanisms of the saponin^+^ compound‐induced hiPSC‐CMs to stabilize electrical signals. WB analysis was used to evaluate the expression of calcium cycle‐related proteins, including SERCA2α, PLB, CAMK II, and Kv1.5 (potassium channel protein) (Figure 4K). Compared to the model group, SERCA2α, and Kv1.5 showed trends of higher expression but significantly lower expression levels of PLB and CAMK II in the intramyocardial Tx group (Figure 4L). Altogether, these data indicated that the saponin^+^ compound‐induced hiPSC‐CMs may improve cardiac performance in severely damaged myocardium by affecting cardiomyocyte integrity, promoting mitochondrial transfer, regulating calcium circulation and ion channel proteins to stabilize electrical activity, and inhibiting myocardial hypertrophy.

### 2.9 Optimally induced hiPSC‐CMs transplantation stimulates the release of growth factors and promotes cardiac repair

**FIGURE 5:**
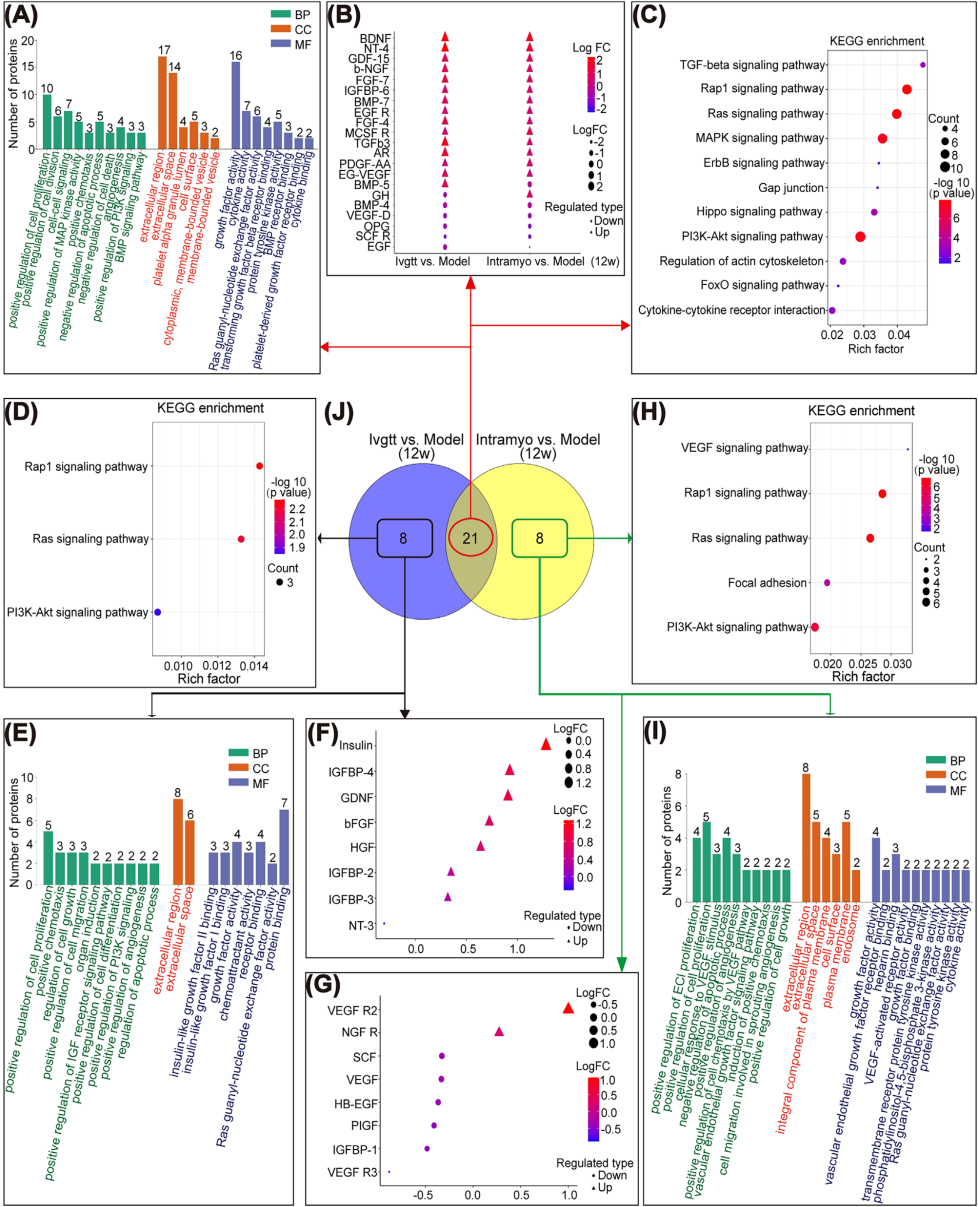
Analysis of the growth‐factor‐related proteins of the peripheral blood using a growth factor antibody array after 12 weeks of hiPSC‐CMs transplantation treatment. (A–C) The intravenous and intramyocardial transplantation groups were compared to the model group, and the common parts of the differential proteins were analyzed by GO and KEGG analyses. (D–F) Among the differential proteins of the intravenous and intramyocardial transplantation groups compared to the model group, the differential proteins unique to the intravenous group but not to the intramyocardial group were analyzed by GO and KEGG analyses. (G–I) Among the differential proteins of the intravenous and intramyocardial transplantation groups compared to the model group, the differential proteins unique to the intramyocardial group but not to the intravenous group were analyzed by GO and KEGG analyses. (J) Venn diagrams of the differentially expressed proteins in the two comparison groups. The number of overlapping proteins > 2‐fold differentially regulated (*p* < 0.001) was determined and mapped. Model_12w, 12 weeks after myocardial infarction modeling; Ivgtt_12w, 12 weeks after the intravenous transplantation of hiPSC‐CMs; Intramyoc_12w, 12 weeks after intramyocardial transplantation of hiPSC‐CMs; Ivgtt vs. Model (12w), differentially expressed proteins in the hiPSC‐CM intravenous transplantation group after 12 weeks compared to the model group; Intramyoc vs. Model (12w), differentially expressed proteins in the hiPSC‐CM intramyocardial transplantation group after 12 weeks compared to the model group. BP, biological process; CC, cellular_component; MF, molecular function.

RNA‐seq analysis indicated that the paracrine process regulated by different routes of hiPSC‐CMs transplantation may be related to the secretion of paracrine mediators, especially growth factors, by the transplanted cells. Thus, we performed a growth factor array analysis to identify paracrine factors that may induce repair mechanisms in injured myocardial tissue. A total of 40 growth factors were detected, and their expression was confirmed in the peripheral blood of macaques after 1, 2, 4, 8, and 12 weeks of hiPSC‐CMs transplantation treatment (Figures S5A–J, S6A–J, S7A–J, and S8A–J; Figure 5A–J). The protein array enrichment analysis of differentially expressed proteins in the Ivgtt versus Model and Myoc versus Model comparisons showed dynamic changes in growth factors at different time points after hiPSC‐CMs transplantation. The signaling pathways jointly regulated by the intravenous and intramyocardial Tx groups were as follows: pathways regulating cardiac development and regeneration (Hippo and ErbB pathways); pathways inhibiting cardiac injury and apoptosis (PI3K‐Akt and MAPK pathways); and pathways impeding pathological myocardial remodeling (Ras and Rap1 pathways). These results suggested multiple paracrine mechanisms through which hiPSC‐CMs transplanted by intravenous and intramyocardial routes may promote cell survival and cardiac repair. Furthermore, we focused on the results of the growth factor array 12 weeks post‐Tx treatment for in‐depth analysis. A Venn diagram constructed with protein sets that were significantly altered in the intravenous and intramyocardial Tx groups identified 21 common differential proteins compared to the model group (Figure 5J). The differential proteins are shown in Figure 5B. GO enrichment analysis showed an overrepresentation of the following components: positive regulation of cell proliferation, cell division, and MAP kinase activity; responding to cell‐cell signaling; sprouting angiogenesis; negative regulation of apoptotic process and cell death; and upregulation of the PI3K signaling pathway (Figure 5A). KEGG analysis indicated that in addition to the common pathways regulated by the other transplantation time points mentioned above, the specific pathways enriched at the 12th‐week post‐Tx also included the FoxO pathway, TGF‐beta pathway, cytokine‐cytokine receptor interaction and gap junction (Figure 5C). We then analyzed the 8 differential proteins unique to the intravenous group (Figure 5F). Although no new regulatory processes or pathways were found in GO and KEGG analyses (Figure 5E,D), we noticed that the key proteins that regulated the activation of related pathways, such as insulin, bFGF, and HGF, were highly expressed in the intravenous Tx group (Figure 5F). Moreover, we also analyzed the 8 differential proteins unique to the intramyocardial group (Figure 5G) and found that activation of the VEGF signaling pathway was unique to the intramyocardial Tx group in GO and KEGG analyses (Figure 5H,I). Thus, the functional benefits associated with hiPSC‐CMs transplantation may be accompanied by the release of growth factors in the peripheral blood of macaques, yet the intravenous and intramyocardial Tx groups had both commonness and uniqueness in the ways of regulating the release of growth factors.

### 2.10 Optimized hiPSC‐CMs transplantation enhances the expression of angiogenic molecules to promote the vasculogenic response to MI injury

**FIGURE 6:**
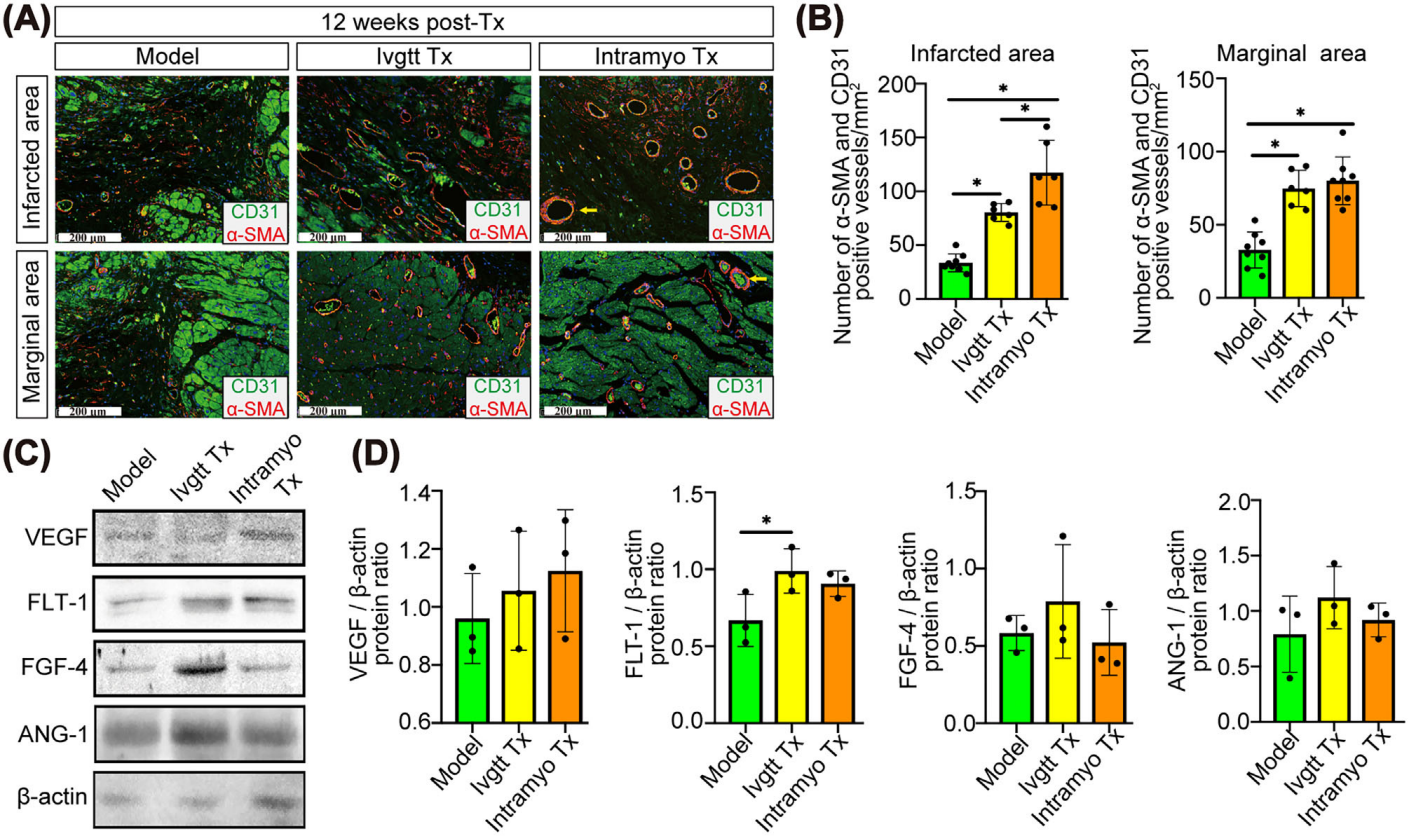
hiPSC‐CMs transplantation enhances the angiogenic response and promotes the expression of angiogenic cytokines after myocardial infarction. (A) Representative micrographs of heart sections from infarcted and marginal areas. The vessel densities (costained with α‐SMA and CD31) were significantly higher in the hiPSC‐CM transplantation groups compared to the model group. The newly formed blood vessels in the intramyocardial transplantation group were more mature with a thicker wall and better integrity, and they showed continuous endothelium and no vascular leakage as indicated by the yellow arrows. Scale bar = 200 μm. (B) Vessel density was determined by counting vascular structures that expressed both α‐SMA and CD31. (C) The growth factors known to enhance angiogenesis in heart tissues, such as VEGF, FLT‐1, FGF‐4, and ANG‐1, were detected by western blotting. (D) The protein expression levels were normalized to the β‐actin levels. All data were represented as the mean ± SD. ^*^ *p* < 0.05 was considered significant. Model: MI‐model group that received no cell transplantation; Ivgtt Tx, intravenous transplantation group; Intramyo Tx, intramyocardial transplantation group.

Based on the RNA‐seq and growth factor array detection results, optimally induced hiPSC‐CMs transplantation was accompanied by the process of angiogenesis, which may contribute to improved cardiac function. Therefore, we assessed whether transplantation of saponin^+^ compound‐induced hiPSC‐CMs promotes angiogenesis in infarcted and marginal areas of MI hearts. Because CD31 labels endothelial cells (ECs) and alpha‐smooth muscle actin (α‐SMA) labels vascular smooth muscle, arteriole, and capillary densities were quantified by α‐SMA and CD31 co‐staining (Figure 6A,B). At 12 weeks after transplantation, arteriolar density was significantly higher in the intramyocardial Tx group than in the intravenous Tx group in the infarcted area of the heart, and both were also significantly higher than that in the model group. Additionally, intravenous and intramyocardial transplantation of hiPSC‐CMs resulted in a robust increase in CD31‐ and α‐SMA‐positive capillary density in the marginal area of MI hearts compared to the model group. However, no significant differences were observed between the intravenous and intramyocardial Tx groups with respect to the marginal zone (Figure 6B). From the perspective of the morphology and structure of neovascularization (Figure 6A), the shape of neovascularization in the myocardium of the intravenous Tx group was irregular, and lack of integrity of the intima‐media membrane. Moreover, the neovascularization in the intramyocardial Tx group had a thick wall (yellow arrow) with a continuous intima. To elucidate the possible mechanisms of different transplantation routes of hiPSC‐CMs on modulating heart function improvements and angiogenesis, the protein expression of cytokines known to promote angiogenesis, including VEGF and FLT‐1, as well as FGF‐4 and ANG‐1, was measured at 12 weeks after cell transplantation. WB results showed that intramyocardial transplantation of hiPSC‐CMs upregulated the expression of three angiogenesis‐related factors, namely, VEGF, FLT‐1, and ANG‐1, but there was a downward trend in FGF‐4 expression compared to the model group. Moreover, the protein expression of VEGF, FLT‐1, FGF‐4, and ANG‐1 increased in the intravenous Tx group (Figure 6C,D). These data suggested that transplantation of hiPSC‐CMs by different routes promoted cardiac repair and neovascularization, probably through inducing paracrine mechanisms, such as augmenting the expression of multiple biological factors associated with enhancing angiogenesis.

## 3 DISCUSSION

Despite the fact that multiple cell‐based transplantation strategies have shown great promise in improving cardiac function, the application of cell therapy faces numerous challenges due to malignant arrhythmia complications and variable cell transplantation routes that affect the quality and capacity for myocardial repair.^17^, ^18^, ^19^ Previously, we established saponin^+^ compound‐induced hiPSC‐CMs with late‐stage fetal heart development physiological characteristics and transplanted them into heart failure mice, demonstrating a protective effect for heart failure therapy.^13^ However, whether it is effective in large animals remains unknown, and systematic comparative studies of multiroute transplantations are required. The current study was the first to assess the safety and efficacy of different saponin^+^ compound‐induced hiPSC‐CMs transplantation routes in a nonhuman primate model. Due to the high demand for the quantity of cardiomyocytes, we prepared a total of six batches of hiPSC‐CMs for transplantation. After transplantation, we used functional and structural pathology methods to comprehensively evaluate cardiac function.

The echocardiographic data showed that intramyocardial Tx improved heart performance in hiPSC‐CMs while intracoronary Tx aggravated cardiac dysfunction. It is worth noting that the protective effect of intravenous Tx varied, indicating that the second enhanced transplantation at 4 weeks can be considered for future use. The Swan‐Ganz catheter^20^ was later utilized to collect precise hemodynamic data, resulting in direct evidence for relieving intracardiac pressure following hiPSC‐CMs transplantation. When the pressure in the cardiac cavity changes, a “metabolic switch” occurs, and energy metabolism shifts from fatty acid metabolism to glucose metabolism.^21^, ^22^ Changes in energy levels may influence subsequent myocardial metabolism and, as a result, contribute to cardiac disease.^23^ According to our PET/CT findings, hiPSC‐CMs therapy encourages energy homeostasis remodeling, which enables the infarcted hearts to resume their normal myocardial state of fatty acid metabolism. Moreover, one of the most critical concerns associated with cardiac cell therapy is the occurrence of tachyarrhythmic complications,^5^, ^24^ when our saponin^+^ compound‐induced hiPSC‐CMs were transplanted, no spontaneous arrhythmias were found, but a slow heart rate was noticed in the intramyocardial Tx group, indicating that the transplanted hiPSC‐CMs may have decreased the heart rate to reduce myocardial oxygen consumption, which has been confirmed that moderate heart rate reduction can promote cardiac regenerative repair after myocardial injury.^25^

Although no teratomas were found during the structural evaluation, renal atrophy and mural thrombosis in the intracoronary Tx group caused the macaques to die before the end of the experiment. Although it has been reported that intracoronary Tx is effective,^26^, ^27^ studies with opposite conclusions have questioned its safety.^28^ Our research on nonhuman primates suggested that when used, the intracoronary Tx strategy necessitates consideration of security risks. Inhibiting the increase in cardiac remodeling and reducing myocardial fibrosis, the intramyocardial route outperforms the intravenous route, these changes are consistent with another study that found positive remodeling after hiPSC‐CMs transplantation.^29^

Strong survival and homing abilities are needed characteristics for an ideal stem cell treatment.^30^ In the current research, transplanted hiPSC‐CMs homed to the injured heart within 4 weeks, despite the fact that the homing cells were few and looked like fragments. Several studies have shown that mitochondrial transfer plays a significant role in cell therapy.^31^, ^32^ Interestingly, we discovered human mitochondria in the myocardium after 4 weeks of hiPSC‐CMs transplantation. We hypothesize that mitochondrial transfer is one of the reasons for the improved heart function, even in the absence of hiPSC‐CMs at weeks 8 or 10. Besides, we discovered that the intramyocardial Tx of hiPSC‐CMs attenuated apoptosis and increases the expression of Nkx2.5 and GATA4 in host cardiomyocytes, both of which are known to protect cardiomyocytes from oxidative damage.^15^, ^16^ Furthermore, we detected the key proteins that affect electrical remodeling, including SERCA2α, PLB, CAMK II, and Kv1.5. SERCA2α and PLB influence cardiomyocyte repolarization and depolarization by regulating diastolic calcium channels, while CAMK II and Kv1.5 activate K^+^ currents and affect cell membrane excitability.^33^, ^34^, ^35^, ^36^ Our data confirmed that intramyocardial Tx inhibited sarcoplasmic reticulum calcium leakage by lowering PLB and CAMK II expression and increasing SERCA2α expression to prevent arrhythmia, and the stimulating effect of intramyocardial Tx based on Kv1.5 may be a potential physiological advantage for regulating myocardial excitability.

Growing evidence indicates that paracrine factors play important roles in cell therapy.^37^ In our research, we discovered that improved expression of FLT‐1, ANG‐1, and VEGF is an important feature of hiPSC‐CMs therapy to enhance neovasculogenesis. The development of neovascularization into mature vessels requires the proliferation and infiltration of ECs, as well as the recruitment and coating of vascular smooth muscle cells and pericytes.^38^ As a major downstream factor mediating angiogenesis, VEGF promotes EC proliferation and migration while also preventing vascular leakage during neovascularization.^39^ Our data showed that the intramyocardial Tx group resulted in more mature neovascularization through the synergistic high expression of VEGF, which is consistent with the phenomenon that the intima‐media was more continuous and complete than that of the intravenous Tx group as observed.

However, there were still several limitations. Because it was a nonhuman primate study, high standard deviations, and small sample size limited our ability to detect statistical significance even though some experiments showed trends. In addition, it can be challenging to maintain consistent quality between batches of hiPSC‐CMs due to manufacturing issues with standardization and quality control. Future large‐scale production of hiPS‐CMs should be accomplished using bioreactors that enable the process through continuous control and stabilization of culture settings to be scalable and reproducible. Moreover, the dosage of our immunosuppressive drugs was based on a macaque immunosuppressive strategy published in *Nature*.^6^ Since several macaques experienced diarrhea during the pre‐experiment due to drug sensitivity, we switched tacrolimus from intramuscular injection to oral administration and paired it with mycophenolate mofetil oral administration to boost efficacy. Even if immune‐deficient animals are selected, it is difficult for transplanted hiPSC‐CMs to engraft into host tissues and survive for an extended period of time.^40^ Thus, we will examine employing a simpler and safer immunosuppressive treatment regimen, such as short‐term oral steroids or cyclosporine,^41^ to help transplanted cells survive and preserve injured hearts during the critical post‐infarction period in our future research. Of particular note, our research indicates a risk of thrombosis and infarction aggravation following coronary delivery of hiPSC‐CMs, but this situation may be avoided by increasing anticoagulation. The heparin used in this study at a dose of 100 U/kg, which is the same as that used in clinical interventional surgery. According to certain reports,^42^, ^43^ when cells are injected through the coronary artery, 400 U/kg of heparin can prevent microvascular obstruction. In the future, we will use enhanced anticoagulation therapy to further validate the safety of hiPSC‐CMs transplantation via a coronary artery.

In conclusion, we conducted the first systematic comparative study on the efficacy and mechanisms of various hiPSC‐CMs transplantation routes and confirmed that saponin^+^ compound‐induced hiPSC‐CMs transplanted via intravenous and intramyocardial routes are safe, which translates to therapeutic efficacy in improving cardiac structural, energy, and electrical remodeling through direct and indirect effects. In terms of the direct process, transplanted cells migrate to the injured myocardium to regulate transcription factor expression, stabilize electrical activity, and inhibit myocardial hypertrophy. Anti‐apoptosis and angiogenesis are aided by an indirect process involving paracrine growth factors. Additionally, the curative effect of intramyocardial transplantation was more stable than that of intravenous transplantation, which may be caused by the difference between the two in the number and integrity of homing cells and the paracrine mechanism. Collectively, these findings support the feasibility of future clinical trials of optimized hiPSC‐CMs as a viable approach for the treatment of acute MI.

## 4 MATERIALS AND METHODS

### 4.1 Human iPS cell culture

The Beijing Saibei Biotechnology Company Ltd. provided hiPSCs, and their pluripotency was verified (Figure S1A). The hiPSCs were maintained in mTeSR medium (STEMCELL Technologies) and cultured on Matrigel (BD Biosciences)‐coated plates; their passage numbers varied from 25 to 40.

### 4.2 Saponin^+^ compound preparation

The saponin^+^ compound is a patent pending product (Chinese pending patent number ZL 202111045654.2) derived from Chinese medicine. Icarin, salvianolic acid B, sphingosine‐1‐phosphate, astragaloside, ginsenoside rg1, PLGF‐2, and a new isomer of scropolioside D with a clearly characterized molecular profile make up the majority of the saponin^+^ compound.^13^ The differentiation and maturation of cardiomyocytes in our system were enhanced by the addition of the saponin^+^ compound at the ideal dosage.

### 4.3 Saponin^+^ compound‐induced cardiomyocyte differentiation and purification

Human iPSCs were grown until 100% confluent on Matrigel (BD Biosciences). For the next 24 h, mTeSR was substituted with CDM medium containing 10 μM CHIR99021 (Sigma‐Aldrich). The medium was then changed to a CDM medium containing 5 ng/ml bFGF (Peprotech) for 24 h, followed by another 24 h of culture in a CDM medium containing the saponin^+^ compound. For the next two days, half of the culture medium was changed with CDM medium containing 5 μM IWP2 (Sigma‐Aldrich) with the saponin^+^ compound. Finally, the differentiated cells were cultured in RPMI 1640/3% KnockOut Serum Replacement (Gibco) medium with the saponin^+^ compound for 48 h before being transferred to a fresh medium. The protocol is shown in Figure S1B. On Days 8, 9, 11, 13, and 15, the medium was replaced with glucose‐free DMEM (Invitrogen) containing 4 mmol/L L‐lactic acids (Sigma) to metabolically select and purify cardiomyocytes. The saponin^+^ compound‐induced hiPSC‐CMs expressed cardiac‐specific markers (Figure S1C), exhibited spontaneous contraction (supporting movie), and were used for transplantation.

### 4.4 Nonhuman primate surgeries and hiPSC‐CMs transplantation

Four‐ to six‐year‐old male macaques, weighing between 7 and 14 kg (purchased from Sichuan Greentech Co., Ltd.), were used as recipients. The characteristics and survival rates of each animal are shown in supplemental Table S1. Macaques first underwent a 2‐week period of acclimation and training to wear a mesh jacket to prevent removal of intravenous (i.v.) catheter (Figure 1A). Before MI creation, an i.v. lidocaine bolus of 1 mg/kg and an infusion of 20 μg/kg/min were used to prevent ventricular arrhythmias. For all major surgeries, macaques were anesthetized with ketamine (15 mg/kg, intramuscularly‐i.m.), intubated with a tracheal tube (4 mm diameter), and maintained on a combination of sevoflurane (2.0‐4.0%) and isoflurane (1.5%–2.0%). Buprenorphine was administered to provide postoperative pain relief. Blood pressure, ECG, and oxygen saturation were monitored during surgery. In brief, a left thoracotomy was performed, and the left anterior descending (LAD) coronary artery was then visualized using a dissecting microscope and subsequently ligated using a polypropylene tube (ligation at 60% of the total length of the artery, measured from the apex end). A silicon tube was placed on top of the polyethylene tube and tied off with a 4.0 silk suture. After 3 h of occlusion of the mid‐LAD coronary artery, the heart was reperfused by removing the tubing. Immune suppression was achieved by daily intramuscular injection of methylprednisolone and maintained at 1 mg/kg until monkeys were euthanized. The monkeys were orally administered mycophenolate mofetil (120 mg/kg/d) and tacrolimus (0.6 mg/kg/d) twice a day with an interval of 12 h, and maintained the serum trough levels of tacrolimus to 10–20 ng/ml, which were then gradually decreased according to the serum concentration.

#### 4.4.1 Intravenous transplantation of hiPSC‐CMs

On the day after MI, the macaque's left arm was stretched and straightened. Intravenous access was established by the elbow vein. A single hiPSC‐CM suspension (1 × 10^8^ hiPSC‐CMs/kg of body weight suspended in 20 ml of sterile normal saline) was intravenously injected at a rate of 4 ml/min (Figure S2A).

#### 4.4.2 Intramyocardial transplantation of hiPSC‐CMs

After MI, the heart of the macaque was exposed. hiPSC‐CMs (10^8^ cells/kg) were delivered intramyocardially into the infarct region and adjacent marginal zones via 10 injections (each 100 μl in volume), using a 29‐gauge injection needle. The syringe was held in place for an additional 3—5 s and then slowly withdrawn. If there was any bleeding, a cotton‐tipped applicator was gently pressed onto the needle insertion site until the bleeding stopped (Figure S2B).

#### 4.4.3 Intracoronary transplantation of hiPSC‐CMs

On the day after MI, followed by a 12 h recovery, heparin (100 U/kg) was delivered i.v. to maintain activated clotting times of 250 s to prevent thrombus formation. The right femoral artery was exposed surgically, and a 4F arterial sheath was inserted using a modified version of the Seldinger technique. Under fluoroscopic guidance (OEC 9800 Plus, GE Medical Systems), a 4F guiding catheter (Cordis, USA) was used to engage the left main coronary artery, and the left coronary arteries were visualized by coronary angiography. Then, a 2.7F microcatheter (Progreat, Terumo, Japan) was superselected into the LAD artery, and angiography confirmed that the microcatheter was accurately positioned. After determining the proper microcatheter position using the radiopaque tip of the microcatheter, the coronary guide wire was pulled out, and a suspension of 10^5^ hiPSC‐CMs (5 ml in volume) was flushed into the LAD artery (Figure S2C). In order to avoid cell overflow, a little contrast agent was pushed into the coronary artery immediately after the cells were injected, so as to flush the cells to the distal coronary artery.

After functional evaluation at the end of the 84‐day survival cycle, the monkeys were euthanized using pentobarbital sodium (concentration = 20 mg/ml; dose = 120 mg/kg body weight) through the intraperitoneal route. Anesthetized animals were euthanized by exsanguination via the abdominal aorta.

### 4.5 Echocardiographic analysis

Baseline transthoracic echocardiography was performed before infarct surgery to evaluate cardiac function. Macaques were sedated with ketamine and propofol, and echocardiography was performed on weeks 1, 2, 4, 8, and 12 posttransplantation. Data were acquired with an ultrasound system with a 5 MHz probe (GE Healthcare, Norway). Transesophageal four‐chamber, two‐chamber, and short‐axis views were collected together with deep transgastric short‐axis views. All measurements were averaged over three consecutive cardiac cycles and analyzed by a single observer who was blinded to the status of the animals.

### 4.6 Intracardiac hemodynamic measurements

A Swan‐Ganz catheter through a 4F sheath placed in the femoral vein guided by fluoroscopy was used for a right heart catheterization. Hemodynamic variables, including CVP, RAP, and RVP, were measured using a hemodynamic monitoring system. All invasive parameters were measured with the anesthetized macaques in the supine position.

### 4.7 PET/CT imaging and analysis

The PET/CT scans were acquired on a dedicated PET/CT scanner (Siemens Biograph 64 Truepoint PET/CT, Germany). This device merges a PET scanner with a multidetector CT scanner and permits the acquisition of coregistered PET and CT images in a single session. Macaques fasted for 6 h before the injection of 3.7 MBq/kg ^18^F‐FDG. Blood glucose (<6 mmol/L) was checked before tracer injection. PET/CT scans were performed after resting for 60 min. A CT scan was obtained initially with a voltage of 120 kV, a current intensity of 60 mA, and a section thickness of 5 mm. PET scans were obtained at 3 min per bed position. PET was performed over the same region immediately after obtaining the CT images. Attenuation correction was performed using CT data, and images were reconstructed as 5 mm slices by applying a standard iterative algorithm. Fused images were converted to DICOM format and analyzed with OsiriX shareware (Geneva, Switzerland) by manually selecting regions of interest (ROIs) of the anterior wall and septal wall of the LV. The SUVmax was calculated using the following formula: SUVmax = radioactivity in ROI (Bq/cm^3^)/injected dose (Bq)/body weight (g). PET/CT images were analyzed by a skilled radiologist and a qualified nuclear medicine physician.

### 4.8 Electrocardiography measurements

All ECGs were obtained (CP150, Welch Allyn, USA) with the anesthetized macaques in dorsal recumbency and the leads attached to the wrists and ankles. All ECGs were interpreted by the same person to reduce interreader variability. The recorded electrocardiographic leads included leads V1‐V6, I, II, III, aVR, aVL, and aVF. Rhythm analysis was conducted by visual inspection.

### 4.9 Histology and immunofluorescence

H&E staining was performed by standard methods using 5 μm paraffin sections. Fibrosis and apoptosis were measured in hearts transplanted with saponin^+^ compound‐induced hiPSC‐CMs by using Masson's Trichrome and TUNEL assays. Immunohistochemical staining was performed on heart tissues after optimized hiPSC‐CMs transplantation. Tissue sections were fixed in 10% formalin and sectioned (5 μm thickness). Briefly, the sections were dewaxed, microwaved to retrieve antigen, blocked with 5% BSA and incubated overnight at 4°C with a primary antibody against Nkx2.5, GATA4, SC121 and AMA. The sections were then hybridized with horseradish peroxidase (HRP)‐labeled secondary antibodies followed by a 3,3’‐diaminobenzidine (DAB) chromogenic reaction. The brown DAB deposits were observed under a microscope (Olympus America Inc., Melville, NY, USA). Immunofluorescence was performed on 5 μm frozen sections using antibodies against α‐SMA and CD31. Sections were blocked, and antibodies were diluted using 1 × casein‐10%‐normal serum‐PBST (Vector Laboratories, Burlingame, CA). Primary antibodies were incubated overnight at 4°C, and secondary antibodies were incubated for 1 h at room temperature. Sections were stained with DAPI during washing, and coverslips were mounted with VECTASHIELD HardSet mounting medium (Vector Laboratories). All antibodies are listed in Table S2.

### 4.10 Transmission electron microscopy

Heart tissue was immediately cut into 1 mm cubes and fixed with 4% glutaraldehyde overnight at 4°C. The tissue was then immersed in 1% osmium tetroxide for 1 h and sectioned, and the semithin sections were stained with toluidine blue. Specimens were then dehydrated in ascending concentrations of ethanol, including en‐bloc contrasting, using 2% uranyl acetate in 70% ethanol for 1 h. The sections were then double stained with uranyl acetate and lead citrate, and they were examined under a LIBRA 120 transmission electron microscope (Carl Zeiss, Germany). All sections were examined and photographed by the same histopathologists.

### 4.11 RNA isolation and RNA‐seq analysis

Macaque hearts were used to extract total RNA, which was then tested using an RNA Nano 6000 Assay Kit and an Agilent 2100 bioanalyzer (Agilent Technologies, CA, USA). Library creation was done using a TruSeq Stranded mRNA Library Prep Kit LT (Illumina, San Diego, CA, USA) in accordance with the manufacturer's instructions for library preparation and sequencing. Using the Agilent Bioanalyzer DNA‐1000 Kit (Agilent Technologies), library quality and size distribution were assessed. The libraries' average sizes were roughly 260 bp, as advised by Illumina. With the use of the Bio‐Rad CFX 96 KIT IQ SYBR GRN, quantitative PCR was carried out to pool equivalent numbers of libraries with various adaptor indices. The NextSeq 500 system (Illumina) was used for all sequencing, and a NextSeq500 mid‐output v2 paired‐end sequencing kit with 150 cycles was used (Illumina).

### 4.12 Growth factor array

The secretory profile of the peripheral blood was analyzed by a growth factor antibody array at 1, 2, 4, 8, and 12 weeks after hiPSC‐CMs transplantation. The analysis of growth factor levels was performed using a Human Growth Factor Array kit (cat# GSH‐GF‐1, RayBiotech Inc., USA) according to the manufacturer's instructions. Briefly, after a 30‐minute incubation at room temperature with sample diluent, the glass chips were washed six times, and each well‐arrayed with human growth factor antibodies was overlaid with 80 μl of the medium. After overnight incubation at 4°C and extensive washing, the detector antibody was added for 1 h and then washed away, and 80 μl of Cy3 equivalent dye‐conjugated streptavidin was added for 1 h at room temperature. The slides were scanned with an InnoScan 300 microarray scanner (Innopsys Parc d'Activités Activestre, Carbonne, France), and signal extraction was performed with InnoScan 300 microarray software. It should be noted that this Human Growth Factor Array kit cannot distinguish between human and monkey‐derived factors and lacks the specificity of human cytokine detection.

### 4.13 Western blot analysis

The isolated MI region from the macaques was homogenized to create protein samples. Each sample of 50 mg heart tissue was lysed and centrifuged in 500 μl of protein RIPA lysis solution from Santa Cruz Biotechnology in California, USA. Equalized tissue lysates were transferred for 1 h to nitrocellulose membranes, where they were separated by electrophoresis using a 10% polyacrylamide gel (Bio‐Rad). With nonfat milk (5%), the membranes were occluded for one hour. Following blocking, primary antibodies against Bcl‐2, Bax, CX43, α‐actinin, c‐TNI, SERCA2α, PLB, CAMK II, Kv1.5, VEGF, FLT‐1, FGF‐4, ANG‐1, and β‐actin were used to probe the membranes. Secondary antibodies that had been coupled to HRP and enhanced chemiluminescence tools were used to identify primary antibody binding. The bands were then scanned, and using β‐actin as the internal control, the expression level of the matching protein in each group was determined. Table S3 contains a list of all antibodies.

### 4.14 Statistical analysis

All statistical analyses were performed using SPSS (IBM SPSS 23.0, SPSS Inc.). A two‐sided Student's t‐test was employed to determine statistical significance for the comparison of two mean values. The pairwise multiple comparison techniques used two‐way analysis of variance testing, followed by Bonferroni t‐tests. A *p‐*value < 0.05 was considered statistically significant.

## AUTHOR CONTRIBUTIONS

Anlong Xu and Hongmei Li conceptualized and designed the study; Data collection: Yuyin Feng, Ting Wang, and Hongmei Li; Hongmei Li and Ke Sun performed the data analysis and interpretation. Statistical analysis: Hongmei Li, Ting Wang; Drafting of the manuscript: Hongmei Li; Critical revision and final approval of the submitted manuscript: Anlong Xu; Obtained funding: Anlong Xu; Technical support: Guangrui Huang and Yulin Cao. All authors have read and approved the final manuscript.

## CONFLICT OF INTEREST STATEMENT

Author Hongmei Li, Guangrui Huang, and Yulin Cao are employees of Beizhong Jingyuan Biotechnology (Beijing) Co. but have no potential relevant financial or non‐financial interests to disclose. The other authors have no conflict of interest.

## ETHICS STATEMENT

All the procedures were performed following the guidelines of the Association for Assessment and Accreditation of Laboratory Animal Care with the approval number of IACUC—B2020016‐P‐02.

## Supporting information



Supporting Information

Supporting Information

## Data Availability

The datasets used and/or analyzed during the current study are available from the corresponding author upon reasonable request.
