# Supplementary material for: Optimal transplantation strategy using human induced pluripotent stem cell‐derived cardiomyocytes for acute myocardial infarction in nonhuman primates
Source: MedComm (2020). 2023 Jun 7;4(3):e289. doi: 10.1002/mco2.289 (PMC10248032; doi:10.1002/mco2.289)
Supplement: Supplementary file 1 — Supporting Information [file MCO2-4-e289-s002.docx]

**TITLE PAGE**

**Title:** Optimal transplantation strategy using hiPSC derived cardiomyocytes for acute myocardial infarction in nonhuman primates

**Authors list:** Hongmei Li (MD)^1, 2^, Ting Wang (PhD)^1^, Yuyin Feng (MS)^1^, Ke Sun (PhD)^1^, Guangrui Huang (PhD)^1, 2^, Yulin Cao (MD)^2, 3^, Anlong Xu (PhD)^1, 4, *^

**Institution information:** ^1^School of Life Science, Beijing University of Chinese Medicine, Beijing; ^2^Beizhong Jingyuan Biotechnology (Beijing) Limited, Beijing; ^3^Tangyi Holdings (Shenzhen) Limited, Shenzhen; ^4^College of Life Sciences, Sun Yat-Sen University, Guangzhou.

^*^**Corresponding author information:** Name: Anlong Xu (PhD), e-mail: xuanlong@bucm.edu.cn

**
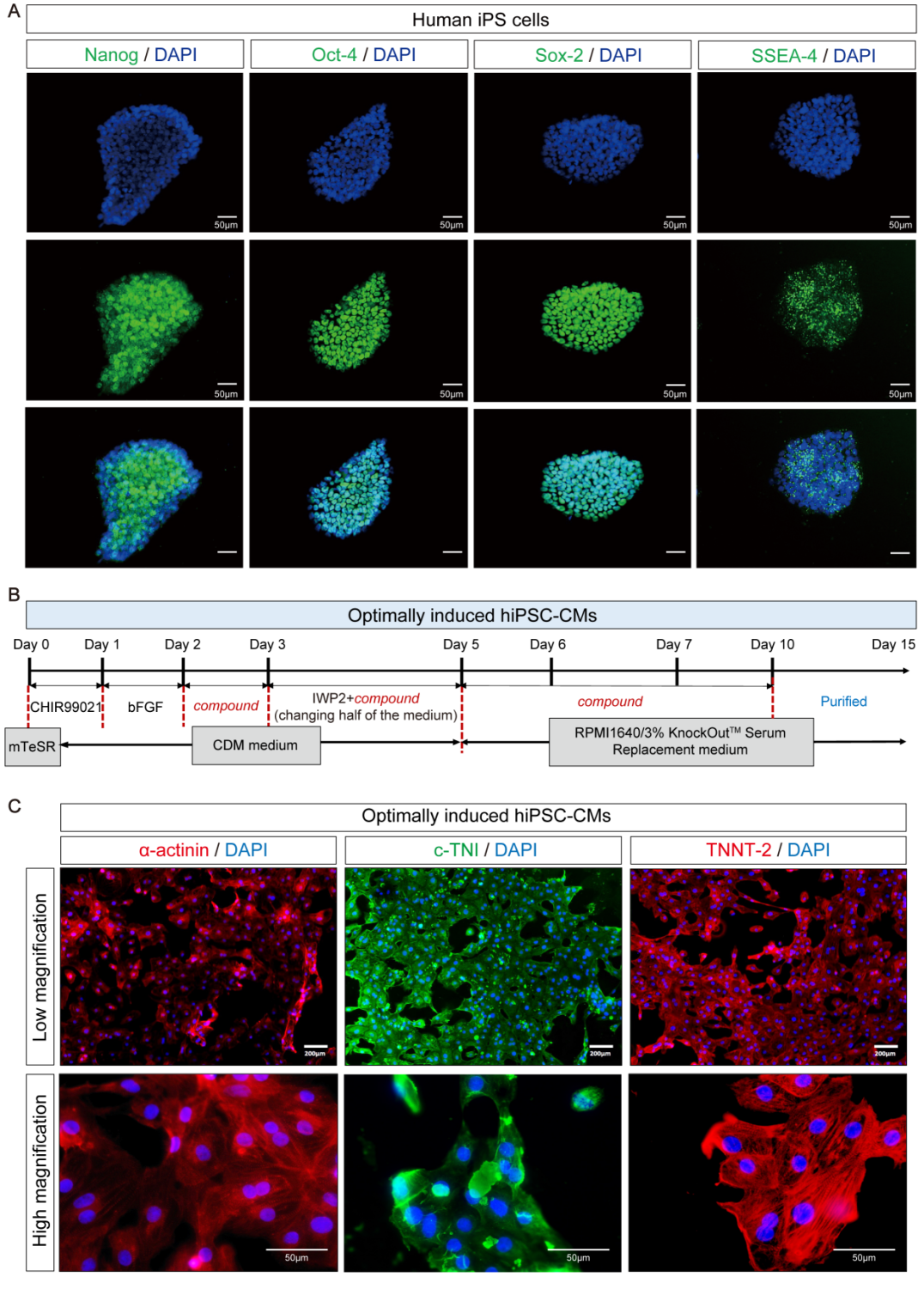
**

**Figure S1. Identification of hiPSCs and saponin^+^ compound-induced hiPSC-CMs.**

**(A)** Immunofluorescent analysis of pluripotency markers (Nanog, Oct-4, Sox-2, SSEA-4). Scale bars: 50 μm. **(B)** Schematic representation of the differentiation procedure (day 0-15) of hiPSC differentiation into cardiomyocytes saponin^+^ compound-induced by effective *saponin^+^* *compound*. hiPSCs, human induced pluripotent stem cells. **(C)** hiPSC-CMs express the cardiac-specific markers including α-actinin, c-TNI and TNNT-2. hiPSC-CMs, cardiomyocytes derived from human induced pluripotent stem cells.


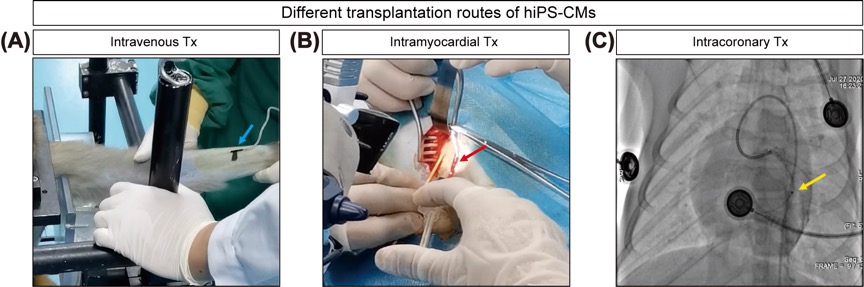


**Figure S2. Technical demonstration of different transplantation routes.**

**(A)** For intravenous transplantation route, the macaques received intravenous infusion (blue arrow) of hiPSC-CMs (1×10^8^ hiPSC-CMs/kg). **(B)** For intramyocardial transplantation route, a suspension of hiPSC-CMs (10^8^ cells/kg) was slowly injected through 10 epicardial puncture sites watching for local tissue blanching indicating myocardial delivery (red arrow). The syringe was held in place for an additional 3 seconds then slowly withdrawn. **(C)** For intracoronary transplantation route, cardiac microcatheter was used to deliver 10^5^ hiPSC-CMs into the left anterior descending branch (yellow arrow).


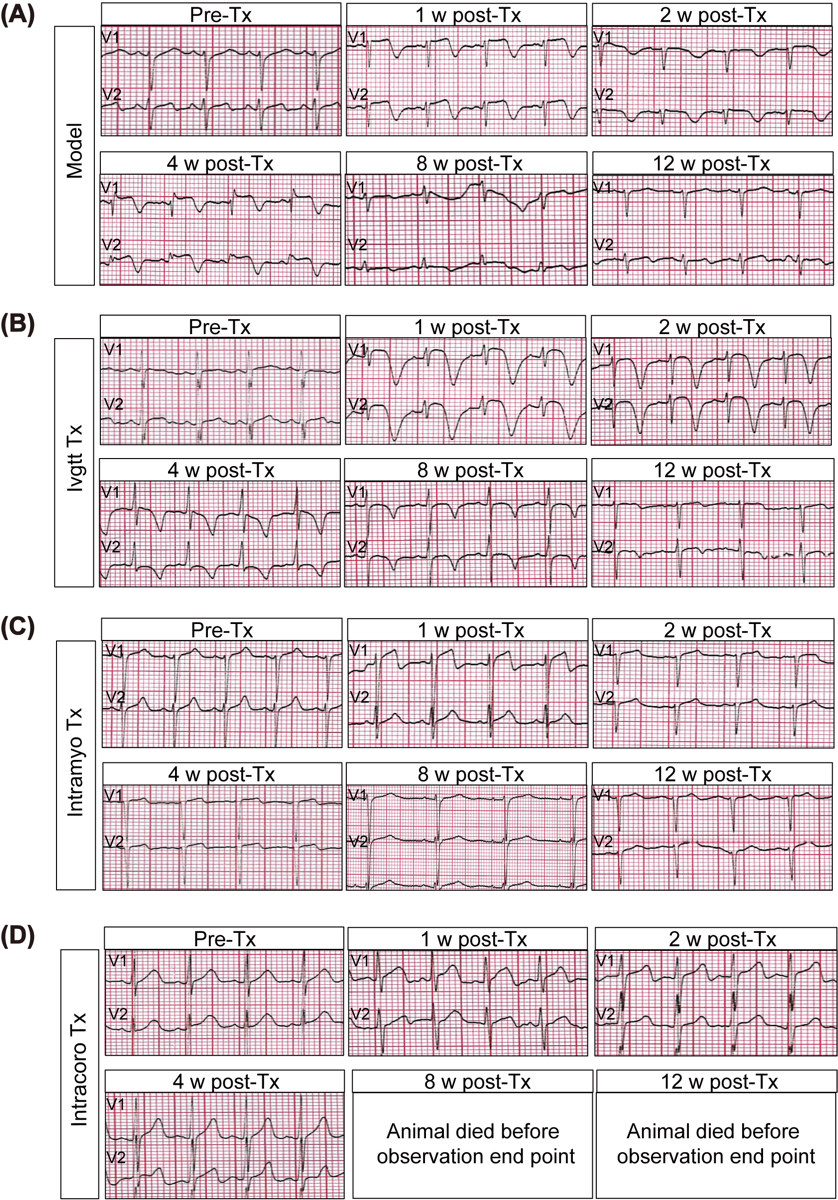


**Figure S3. Effects of the saponin^+^ compound-induced hiPSC-CMs transplanted through different routes on electrical stability.**

**(A-D)** ECG was performed at different time points (before transplantation and 1, 2, 4, 8 and 12 weeks posttransplantation). Representative traces from macaque ECG recordings were shown. Model: MI-model group that received no cell transplantation; Ivgtt Tx, intravenous transplantation group; Intramyo Tx, intramyocardial transplantation group; Intracoro, intracoronary transplantation group.


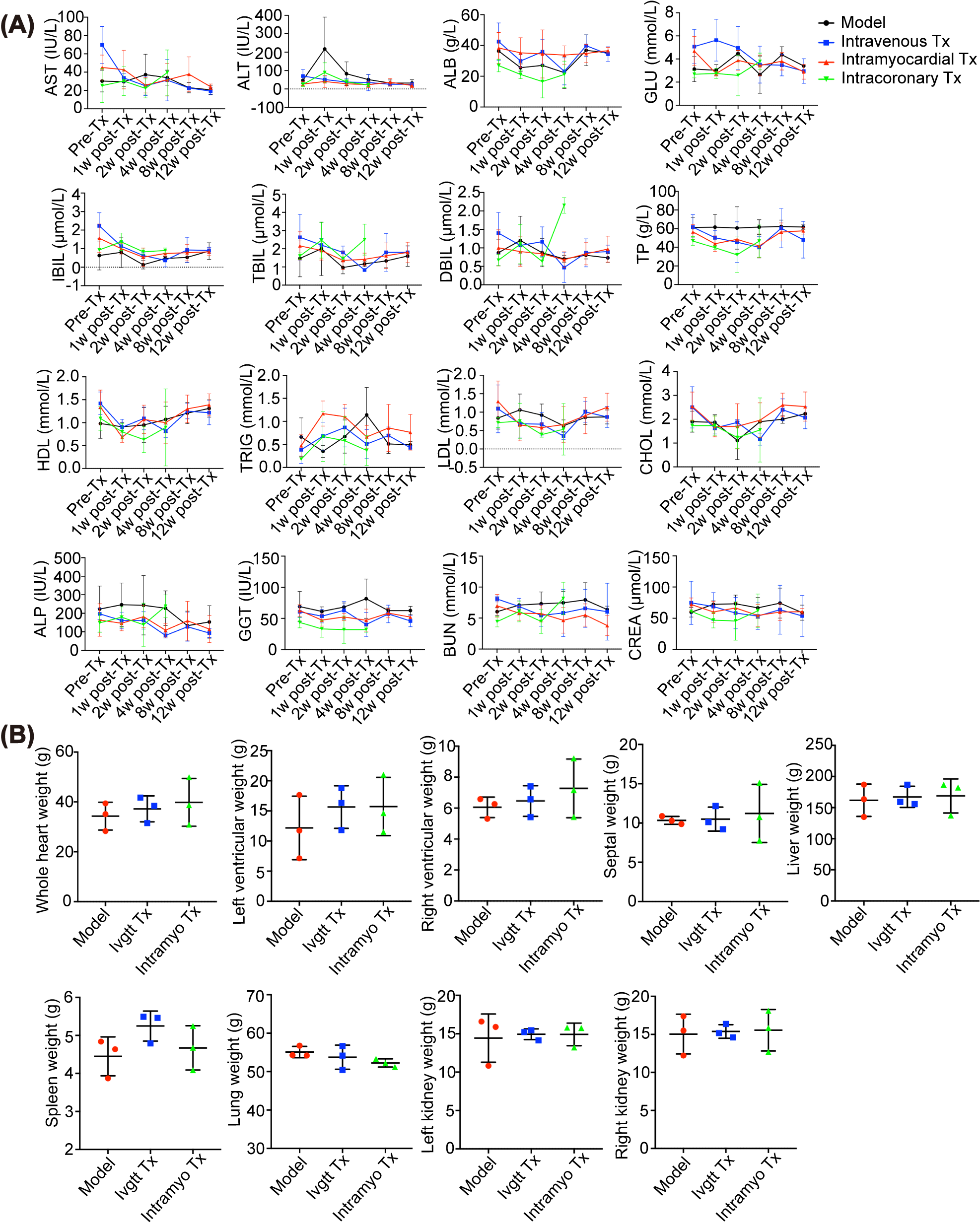


**Figure S4. Biochemical safety index detection and organ weight comparison post-transplantation.**

**(A)** Peripheral blood was collected for liver and kidney function biomarkers assessment. n = 3-5 macaques per group. AST: aspartate aminotransferase; ALT: alanine aminotransferase; ALB: albumin; GLU: glucose; IBIL: indirect bilirubin; TBIL: total bilirubin; DBIL: direct bilirubin; TP: total protein; HDL: high-density lipoprotein; TRIG: triglycerides; LDL: low-density lipoprotein; CHOL: cholesterol; ALP: alkaline phosphatase; GGT: gamma glutamyl transpeptidase; BUN: blood urea nitrogen; CREA: creatinine. **(B)** Organ weight after 12 weeks of hiPSC-CMs transplantation. Data were represented as mean ± SEM (n=3 per group). Whole heart, left ventricular, right ventricular, septal, liver, spleen, lung and kidney organ weight did not significantly differ between model groups. Model: MI-model group that received no cell transplantation; Ivgtt Tx, intravenous transplantation group; Intramyo Tx, intramyocardial transplantation group.

**
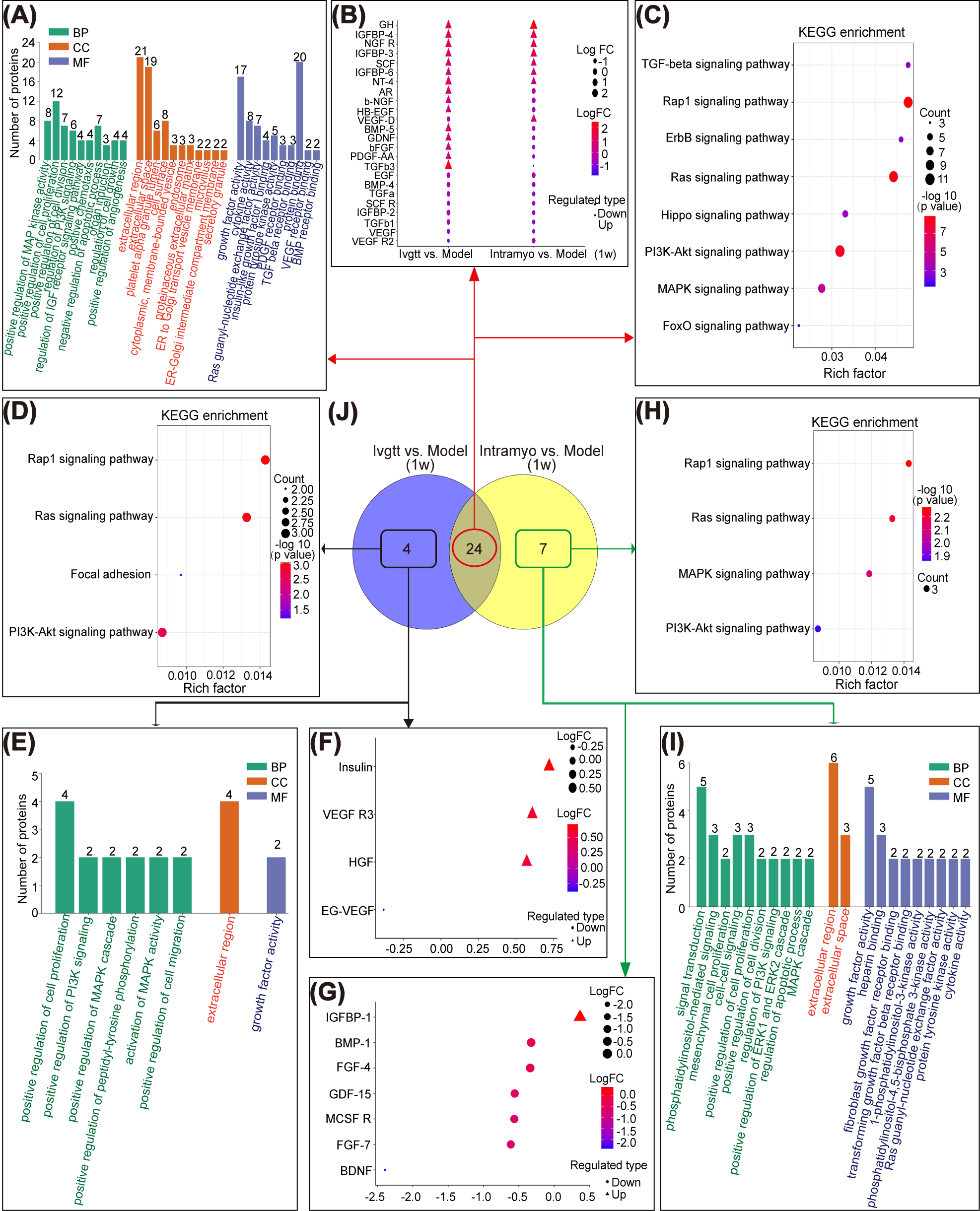
Figure S5. The growth-factor-related proteins of the peripheral blood were analyzed by a growth factor antibody array after 1 week of hiPSC-CMs transplantation treatment.**

**(A-C)** The intravenous and intramyocardial transplantation groups were compared with the model group, and the common parts of the differential proteins were analyzed by GO and KEGG. **(D-F)** Among the differential proteins of intravenous and intramyocardial transplantation groups compared with the model group, the differential proteins unique to the intravenous group but not in the intramyocardial group were analyzed by GO and KEGG. **(G-I)** Among the differential proteins of intravenous and intramyocardial transplantation groups compared with the model group, the differential proteins unique to the intramyocardial group but not in the intravenous group were analyzed by GO and KEGG. **(J)** Venn diagram illustrations of proteins found to be differentially expressed in two comparison groups. The number of overlapping proteins >2-fold differentially regulated (*P*<0.001) was determined and mapped. Model_1w, 1 week after myocardial infarction modeling; Ivgtt_1w, 1 week after the intravenous transplantation of hiPSC-CMs; Intramyoc_1w, 1 week after intramyocardial transplantation of hiPSC-CMs; Ivgtt vs. Model (1w), differentially expressed proteins in the hiPSC-CM intravenous transplantation group after 1 week compared to the model group; Intramyoc vs. Model (1w), differentially expressed proteins in the hiPSC-CM intramyocardial transplantation group after 1 week compared to the model group. BP, biological process; CC, cellular_component; MF, molecular function.

**
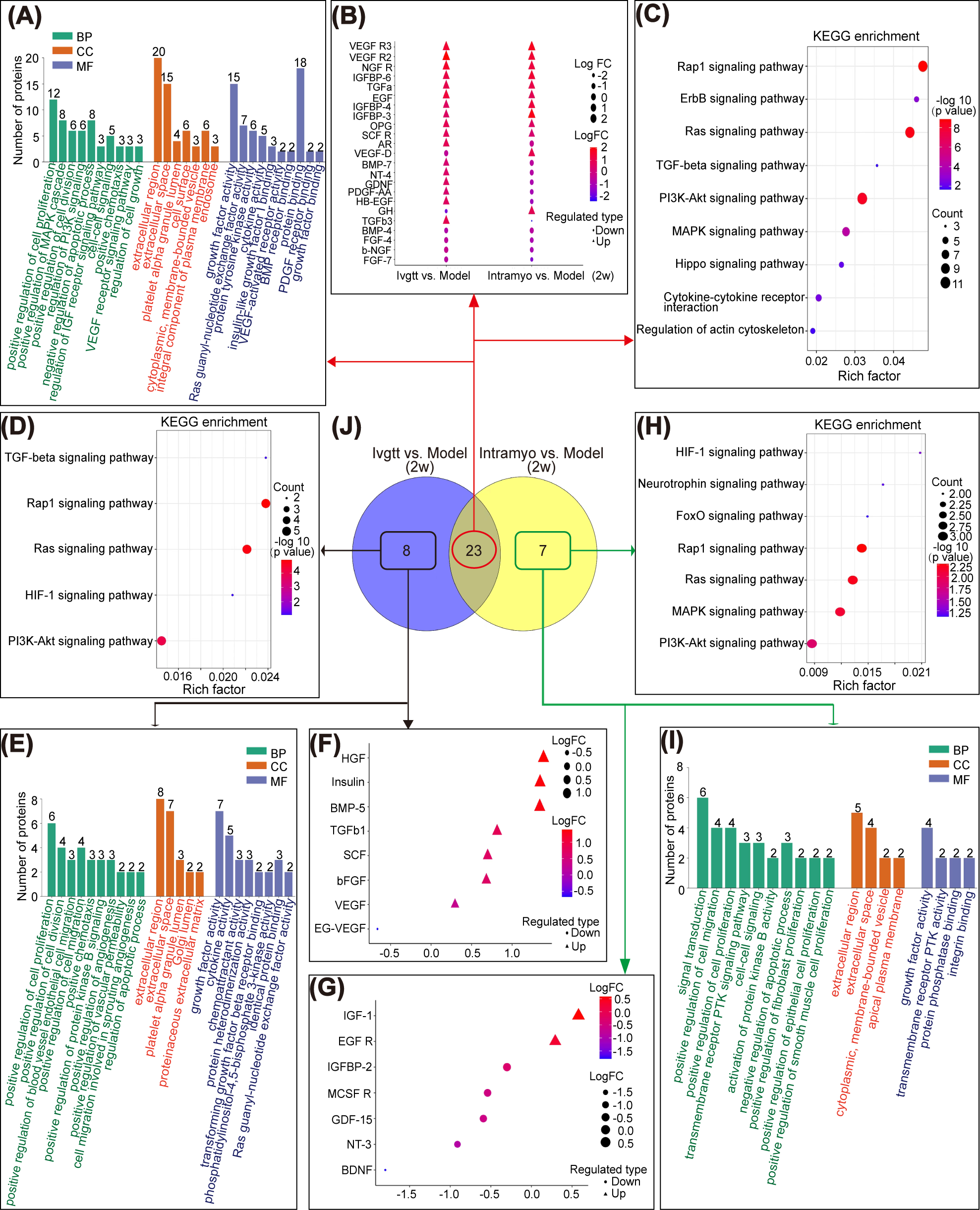
Figure S6. The growth-factor-related proteins of the peripheral blood were analyzed by a growth factor antibody array after 2 weeks of hiPSC-CMs transplantation treatment.**

**(A-C)** The intravenous and intramyocardial transplantation groups were compared with the model group, and the common parts of the differential proteins were analyzed by GO and KEGG. **(D-F)** Among the differential proteins of intravenous and intramyocardial transplantation groups compared with the model group, the differential proteins unique to the intravenous group but not in the intramyocardial group were analyzed by GO and KEGG. **(G-I)** Among the differential proteins of intravenous and intramyocardial transplantation groups compared with the model group, the differential proteins unique to the intramyocardial group but not in the intravenous group were analyzed by GO and KEGG. **(J)** Venn diagram illustrations of proteins found to be differentially expressed in two comparison groups. The number of overlapping proteins >2-fold differentially regulated (*P*<0.001) was determined and mapped. Model_2w, 2 weeks after myocardial infarction modeling; Ivgtt_2w, 2 weeks after the intravenous transplantation of hiPSC-CMs; Intramyoc_2w, 2 weeks after intramyocardial transplantation of hiPSC-CMs; Ivgtt vs. Model (2w), differentially expressed proteins in the hiPSC-CM intravenous transplantation group after 2 weeks compared to the model group; Intramyoc vs. Model (2w), differentially expressed proteins in the hiPSC-CM intramyocardial transplantation group after 2 weeks compared to the model group. BP, biological process; CC, cellular_component; MF, molecular function.

**
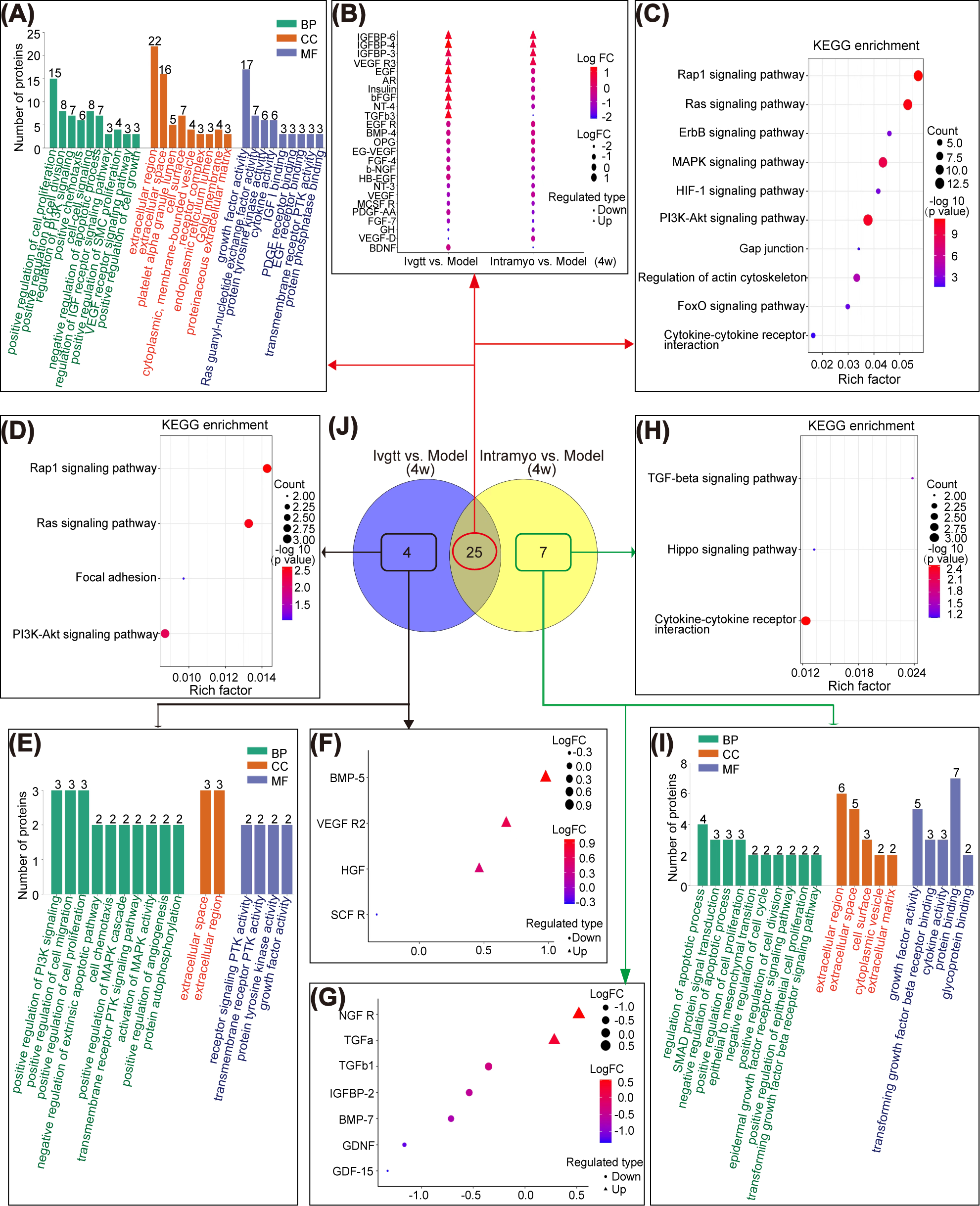
Figure S7. The growth-factor-related proteins of the peripheral blood were analyzed by a growth factor antibody array after 4 weeks of hiPSC-CMs transplantation treatment.**

**(A-C)** The intravenous and intramyocardial transplantation groups were compared with the model group, and the common parts of the differential proteins were analyzed by GO and KEGG. **(D-F)** Among the differential proteins of intravenous and intramyocardial transplantation groups compared with the model group, the differential proteins unique to the intravenous group but not in the intramyocardial group were analyzed by GO and KEGG. **(G-I)** Among the differential proteins of intravenous and intramyocardial transplantation groups compared with the model group, the differential proteins unique to the intramyocardial group but not in the intravenous group were analyzed by GO and KEGG. **(J)** Venn diagram illustrations of proteins found to be differentially expressed in two comparison groups. The number of overlapping proteins >2-fold differentially regulated (*P*<0.001) was determined and mapped. Model_4w, 4 weeks after myocardial infarction modeling; Ivgtt_4w, 4 weeks after the intravenous transplantation of hiPSC-CMs; Intramyoc_4w, 4 weeks after intramyocardial transplantation of hiPSC-CMs; Ivgtt vs. Model (4w), differentially expressed proteins in the hiPSC-CM intravenous transplantation group after 4 weeks compared to the model group; Intramyoc vs. Model (4w), differentially expressed proteins in the hiPSC-CM intramyocardial transplantation group after 4 weeks compared to the model group. BP, biological process; CC, cellular_component; MF, molecular function.

**
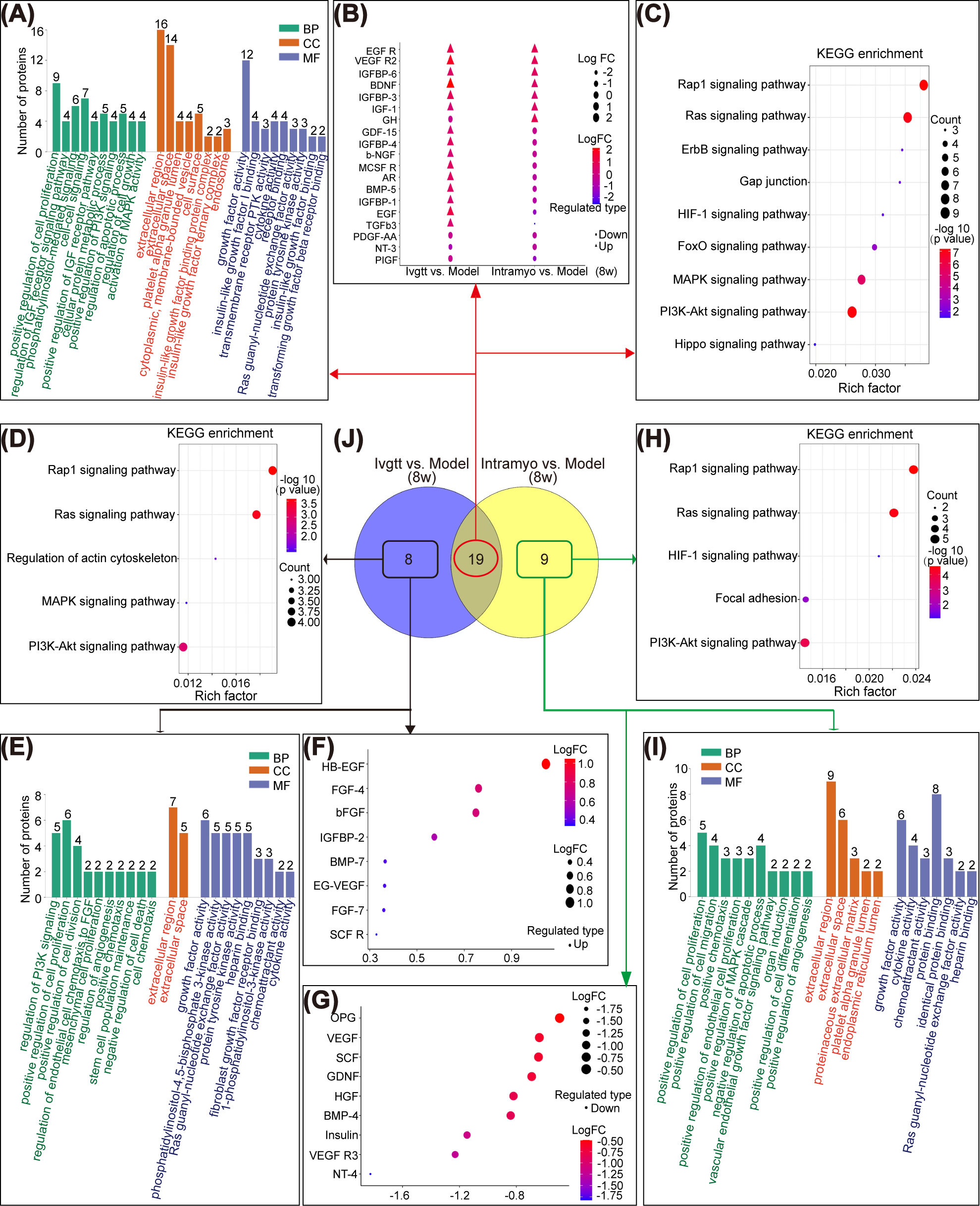
Figure S8. The growth-factor-related proteins of the peripheral blood were analyzed by a growth factor antibody array after 8 weeks of hiPSC-CMs transplantation treatment.**

**(A-C)** The intravenous and intramyocardial transplantation groups were compared with the model group, and the common parts of the differential proteins were analyzed by GO and KEGG. **(D-F)** Among the differential proteins of intravenous and intramyocardial transplantation groups compared with the model group, the differential proteins unique to the intravenous group but not in the intramyocardial group were analyzed by GO and KEGG. **(G-I)** Among the differential proteins of intravenous and intramyocardial transplantation groups compared with the model group, the differential proteins unique to the intramyocardial group but not in the intravenous group were analyzed by GO and KEGG. **(J)** Venn diagram illustrations of proteins found to be differentially expressed in two comparison groups. The number of overlapping proteins >2-fold differentially regulated (*P*<0.001) was determined and mapped. Model_8w, 8 weeks after myocardial infarction modeling; Ivgtt_8w, 8 weeks after the intravenous transplantation of hiPSC-CMs; Intramyoc_8w, 8 weeks after intramyocardial transplantation of hiPSC-CMs; Ivgtt vs. Model (8w), differentially expressed proteins in the hiPSC-CM intravenous transplantation group after 8 weeks compared to the model group; Intramyoc vs. Model (8w), differentially expressed proteins in the hiPSC-CM intramyocardial transplantation group after 8 weeks compared to the model group. BP, biological process; CC, cellular_component; MF, molecular function.

**Table S1. Characteristics of macaques used in the study.**

| **ID** | **Sex** | **Age**  **(year)** | **Weight**  **(kg)** | **Treatment** | **Endpoint** | **Survival** | **Survival rate** |
| --- | --- | --- | --- | --- | --- | --- | --- |
| Model-1 | Male | 4 | 7.75 | No-cell control | 12 weeks (model) | 12 weeks | 100% |
| Model-2 | Male | 4 | 8.10 | No-cell control | 12 weeks (model) | 12 weeks |  |
| Model-3 | Male | 3 | 7.20 | No-cell control | 12 weeks (model) | 12 weeks |  |
| Ivgtt-1 | Male | 3 | 10.45 | Intravenous Tx of hiPSC-CMs | 12 weeks (cells) | 12 weeks | 100% |
| Ivgtt-2 | Male | 4 | 7.10 | Intravenous Tx of hiPSC-CMs | 12 weeks (cells) | 12 weeks |  |
| Ivgtt-3 | Male | 4 | 9.80 | Intravenous Tx of hiPSC-CMs | 8 weeks (cells) | 8 weeks |  |
| Ivgtt-4 | Male | 3 | 8.40 | Intravenous Tx of hiPSC-CMs | 12 weeks (cells) | 12 weeks |  |
| Ivgtt-5 | Male | 4 | 9.80 | Intravenous Tx of hiPSC-CMs | 4 weeks (cells) | 4 weeks |  |
| Intramyo-1 | Male | 4 | 7.85 | Intramyocardial Tx of hiPSC-CMs | 8 weeks (cells) | 8 weeks | 100% |
| Intramyo-2 | Male | 4 | 7.80 | Intramyocardial Tx of hiPSC-CMs | 8 weeks (cells) | 8 weeks |  |
| Intramyo-3 | Male | 4 | 7.60 | Intramyocardial Tx of hiPSC-CMs | 12 weeks (cells) | 12 weeks |  |
| Intramyo-4 | Male | 4 | 9.55 | Intramyocardial Tx of hiPSC-CMs | 12 weeks (cells) | 12 weeks |  |
| Intramyo-5 | Male | 4 | 10.30 | Intramyocardial Tx of hiPSC-CMs | 12 weeks (cells) | 12 weeks |  |
| Intramyo-6 | Male | 4 | 7.60 | Intramyocardial Tx of hiPSC-CMs | 4 weeks (cells) | 4 weeks |  |
| Intracoro-1 | Male | 4 | 8.65 | Intracoronary Tx of hiPSC-CMs | 12 weeks (cells) | 12 weeks | 33.33% |
| Intracoro-2 | Male | 6 | 13.35 | Intracoronary Tx of hiPSC-CMs | 12 weeks (cells) | 4 weeks |  |
| Intracoro-3 | Male | 6 | 10.00 | Intracoronary Tx of hiPSC-CMs | 12 weeks (cells) | 4 weeks |  |

**Table S2. Antibodies used in immunofluorescence and immunohistochemistry analyses.**

| **Target** | **Host** | **Supplier** | **Cat no.** | **Dilution** |
| --- | --- | --- | --- | --- |
| α-SMA | Rabbit | Proteintech | 55135-1-AP | 1:200 |
| CD31 | Mouse | Abcam | ab9498 | 1:200 |
| Nkx2.5 | Rabbit | Novus | NBP2-24675 | 5 ug/ml |
| GATA4 | Rabbit | Proteintech | 19530-1-AP | 1:200 |
| SC121 | Mouse | Cellartis | Y40410 | 1:1000 |
| AMA (working fluid) | Mouse | ZSGB-BIO | ZM-0350 | 1:1 |
| α-actinin | Rabbit | Proteintech | 11313-2-AP | 1:200 |
| TNNT-2 | Mouse | Abcam | ab8295 | 1:200 |
| Nanog | Mouse | Abcam | ab173368 | 1:200 |
| Oct-4 | Mouse | Abcam | ab184665 | 1:200 |
| Sox-2 | Rabbit | Abcam | ab92494 | 1:200 |
| SSEA-4 | Mouse | Abcam | ab16287 | 1:100 |
| Goat Anti-Rabbit IgG H&L (HRP) | Goat | Abcam | Ab205718 | 1:5000 |
| Goat Anti-Mouse IgG H&L (HRP) | Goat | Abcam | Ab205719 | 1:5000 |

**Table S3. Antibodies used in western blot analyses.**

| **Target** | **Host** | **Supplier** | **Cat no.** | **Dilution** |
| --- | --- | --- | --- | --- |
| Bcl-2 | Mouse | Novus | NBP2-29630 | 1:5000 |
| β-actin | Mouse | Affinity | T0022 | 1:1000 |
| β-actin | Rabbit | Affinity | AF7018 | 1:1000 |
| Bax | Rabbit | Cell signaling | 2772 | 1:1000 |
| CX43 | Rabbit | Abcam | ab217676 | 1:5000 |
| α-actinin | Rabbit | Proteintech | 11313-2-AP | 1:10000 |
| c-TNI | Mouse | Proteintech | 66376-1-lg | 1:5000 |
| SERCA2α | Mouse | Proteintech | 67248-1-1g | 1:5000 |
| PLB | Rabbit | Abcam | ab15000 | 1:5000 |
| CAMK II | Rabbit | Proteintech | 15443-1-AP | 1:5000 |
| Kv1.5 | Mouse | Abcam | ab184691 | 1:5000 |
| VEGF | Mouse | Biotechne | 26503 | 1:5000 |
| FLT-1 | Rabbit | Proteintech | 13687-1-AP | 1:5000 |
| FGF-4 | Rabbit | Abcam | ab65974 | 1:5000 |
| ANG-1 | Rabbit | Proteintech | 23302-1-AP | 1:1000 |

**Supplementary movie. Spontaneous pulsation properties of compound saponin^+^ compound-induced hiPSC-CMs on day 15 of differentiation.**

Superiority of contractility and frequency of the saponin^+^ compound-induced hiPSC-CMs are clearly observed. The contraction rate of the spontaneously beating cardiomyocytes is evaluated around 85 contractions/min under compound induction condition. It can be seen from the video that the saponin^+^ compound-induced hiPSC-CMs beat in sheets and waves, and almost all of them can actively beat with strong contractions, and the beating state is very similar to that of normal human cardiomyocytes.
